# Shielded goethite catalyst that enables fast water dissociation in bipolar membranes

**DOI:** 10.1038/s41467-020-20131-1

**Published:** 2021-01-04

**Authors:** Muhammad A. Shehzad, Aqsa Yasmin, Xiaolin Ge, Zijuan Ge, Kaiyu Zhang, Xian Liang, Jianjun Zhang, Geng Li, Xinle Xiao, Bin Jiang, Liang Wu, Tongwen Xu

**Affiliations:** 1 CAS Key Laboratory of Soft Matter Chemistry, Collaborative Innovation Centre of Chemistry for Energy Materials, Department of Applied Chemistry, School of Chemistry and Materials Science, University of Science and Technology of China, 230026 Hefei, China; 2 Advanced Materials and Membranes Technology Centre, Department of Polymer and Process Engineering, University of Engineering and Technology Lahore, G.T. Road, Punjab, 54890 Pakistan; 3 Department of Chemical Physics, School of Chemistry and Materials Science, University of Science and Technology of China, 230026 Hefei, China

**Keywords:** Chemical engineering, Materials for energy and catalysis

## Abstract

Optimal pH conditions for efficient artificial photosynthesis, hydrogen/oxygen evolution reactions, and photoreduction of carbon dioxide are now successfully achievable with catalytic bipolar membranes-integrated water dissociation and in-situ acid-base generations. However, inefficiency and instability are severe issues in state-of-the-art membranes, which need to urgently resolve with systematic membrane designs and innovative, inexpensive junctional catalysts. Here we show a shielding and in-situ formation strategy of fully-interconnected earth-abundant goethite Fe^+3^O(OH) catalyst, which lowers the activation energy barrier from 5.15 to 1.06 eV per HO − H bond and fabricates energy-efficient, cost-effective, and durable shielded catalytic bipolar membranes. Small water dissociation voltages at limiting current density (U_LCD_: 0.8 V) and 100 mA cm^−2^ (U_100_: 1.1 V), outstanding cyclic stability at 637 mA cm^−2^, long-time electro-stability, and fast acid-base generations (H_2_SO_4_: 3.9 ± 0.19 and NaOH: 4.4 ± 0.21 M m^−2^ min^−1^ at 100 mA cm^−2^) infer confident potential use of the novel bipolar membranes in emerging sustainable technologies.

## Abstract (web summary)

Bipolar membranes integrated water dissociation and acid-base generations have great potential in emerging sustainable technologies but remains inefficient. Here, the authors circumvent this inefficiency and instability of the membranes by developing polyaniline shielded catalytic bipolar membranes.

## Introduction

Sustainable advanced processes such as artificial photosynthesis (AP), hydrogen evolution reaction (HER), oxygen evolution reaction (OER) and CO_2_ photoreduction require highly controlled single pH media for their best performance, which is practically unachievable with the conventional ex-situ acid–base supplies^1–5^. This perceptibly insoluble dilemma of achieving long-term optimal pH conditions is solely solvable with catalytic bipolar membranes (CBMs)-integrated in-situ acid–base generations^4^. Wherein a CBM, which comprises a water dissociation (WD) catalyst at the junction of cation–anion exchange layers (CEL|Cat| AEL), continuously intakes water into the catalytic junction and dissociates the feedwater into H^+^/OH^−^ ion pairs^6^. The applied reverse bias releases the produced H^+^/OH^−^ ions from the catalyst surface. It also drives the ions toward the counter electrodes by passing through the C/AEL pair, where they react with their counter-ions to in-situ produce acid and base at the same rate as their consumption in AP, HER, OER and CO_2_ reduction processes and retain the system catalysts at their optimal activity^4,7^. Thus, the integration of CBMs in emerging sustainable technologies can provide optimised architectures to achieve their utmost performance efficiency^4,8,9^. Besides, the CBMs-integrated WD can also possess great potential for numerous industrial processes to treat concentrated salt solutions^10,11^, continuous regeneration of ion-exchange resins during water deionizing process^12^, conversions of lactate into lactic acid and neutralisation of fermentation broth in fermentation reactors^13,14^.

In all these processes, a CBM works as an electrochemical membrane reactor which can efficiently dissociate water and simultaneously separate the reaction products to in-situ produce acid and base. The catalytic junction (reaction zone) in a CBM is of supreme importance, and the water dissociation rate mainly depends on the efficiency of junctional catalysts^7^. Since the invention of CBMs in the 1950s, several junctional catalysts such as carboxylates, silanes, PEDOTs, BiOCl, FeMIL-101-NH_2,_ P.E.I., lysozyme, graphene oxide, MOFs, bovine serum, TiOH, ZrOH, RCOONa, metal oxides and organic–inorganic mixtures have been trialled. These catalysts typically improved the water dissociation rate to some extent (lowered the over-potential voltage, see Supplementary Table 6). However, inefficiency and instability of these catalysts-integrated bipolar membranes precluding their utility in advanced technologies and industrial applications, thus dictate the need for novel junctional catalysts to overcome the current challenges in the CBMs. Previously, much of the research is focused on just the fabrication of CBMs via the spray-blending of these catalysts within the junction, and relatively little attention has been paid in controlling the junctional morphologies, the precision of which is critical to achieving a systematic water dissociation for a robust and stable process.

**Fig. 1:**
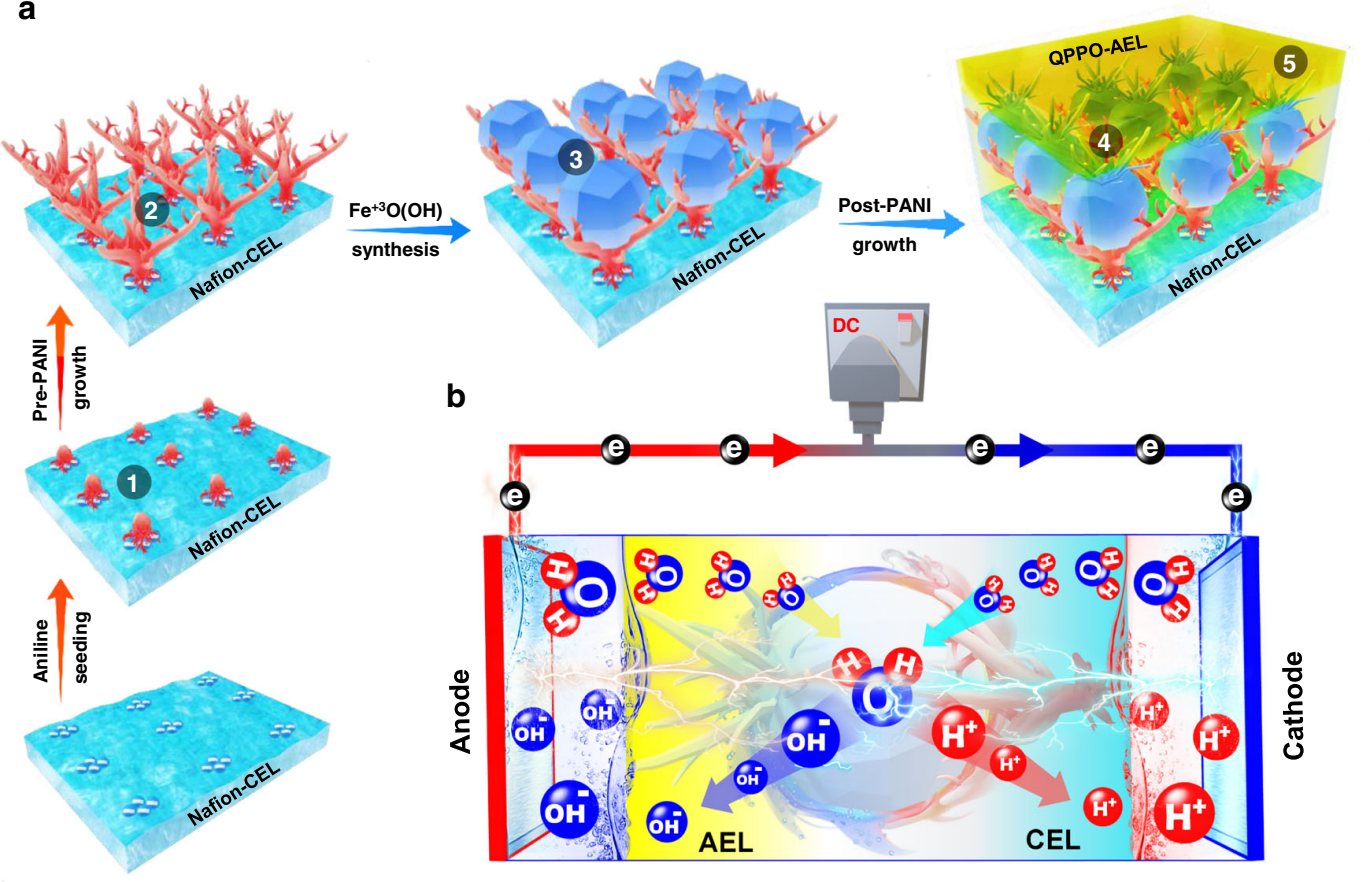
Simplistic SCBM formation route and water dissociation mechanism. **a** Schematic showing the four in-situ chemical steps such as ANI seeding (Stage 1), growth of pre-PANI shield (Stage 2), synthesis of the catalyst nanoparticles (Stage 3), growth of post-PANI shield (Stage 4) and the fifth step for physical infusion of QPPO solution into the catalytic junction to form AEL (Stage 5) sequentially construct an SCBM. **b** An illustration exhibiting diffusion of water molecules through the cyan CEL and yellow AEL into the catalytic junction, wherein the applied reverse bias electric potential dissociating the feedwater and bringing the produced proton-hydroxide ions out of the catalytic junction.

Here we show synthesis of a fully interconnected goethite Fe^+3^O(OH) junctional catalyst for CBMs and first report its excellent catalytic activity using density functional theory (DFT) calculations. We also report a systematic catalyst shielding strategy with heterogeneously in-situ grown electronically conductive polyaniline (PANI) to produce durable CBMs for their confident use in emerging sustainable technologies. Briefly, the in-situ synthesis and catalyst shielding strategies in the proposed route (Stage 1–4 in Fig. 1a), such as aniline (ANI) seeding, growth of pre-PANI shield, catalyst formation and post-PANI shield are crucial to constructing electrochemically ultra-active membrane junction (find details in the following sections). Briefly, the initial aniline (ANI) seeding enables electrostatically entrenched heterogeneous growth of PANI nanostructures at the Nafion-CEL surface^15^. The in-situ grown pre- and post-PANI porous films, which act as adhesion layers, jointly also enable very uniform distribution of the goethite Fe^+3^O(OH) catalyst within the junction and prevent direct contact of the catalyst particles to the CEL^16^. This contact breaking strategy can circumvent the out-migration of metal ions from the membrane junction and enables stable WD performance under an applied electric field^17^. Therefore, the PANI films are hereby named as “shields” due to their catalyst immobilising/protecting characteristics. This immobilisation strategy is particularly useful in the case of metals-based junctional catalysts. Thus, the in-situ synthesis of the goethite Fe^+3^O(OH) catalyst within the PANI shields is of supreme importance in boosting the rate of consistent water dissociation in bipolar membranes. Physico-crystallo-electrochemical analysis with SEM, AFM, X-ray diffraction (XRD), XPS, DFT, EIS and *I*–*V* are performed in detail to theoretically and experimentally validate the energy-efficient water dissociation using the shielded goethite Fe^+3^O(OH) catalytic bipolar membranes (hereafter denoted as shielded catalytic bipolar membranes (SCBMs)). The novel goethite Fe^+3^O(OH) catalyst enables WD at 1.06 eV per OH–H bond (uncatalyzed WD energy barrier is 5.15 eV) and the catalyst shielding with heterogeneously in-situ grown polyaniline improves durability of the bipolar membranes. Therefore, the resulting SCBMs show quick start-up and energy-efficient water dissociation (*U*_LCD_: 0.8 V and *U*_100_: 1.1 V) as well as excellent cyclic and long-time electro-stability. Besides, the SCBMs in an electrodialysis single-cell also show ultrafast in-situ acid–base generations (H_2_SO_4_: 3.9 ± 0.19 M m^−2^ min^−1^ and NaOH: 4.4 ± 0.21 M m^−2^ min^−1^ at 100 mA cm^−2^), thus endorsing the effective and confident use of novel SCBMs in emerging sustainable technologies.

## Results

### Evidence for in-situ synthesis and shielding of Fe^+3^O(OH)

**Fig. 2:**
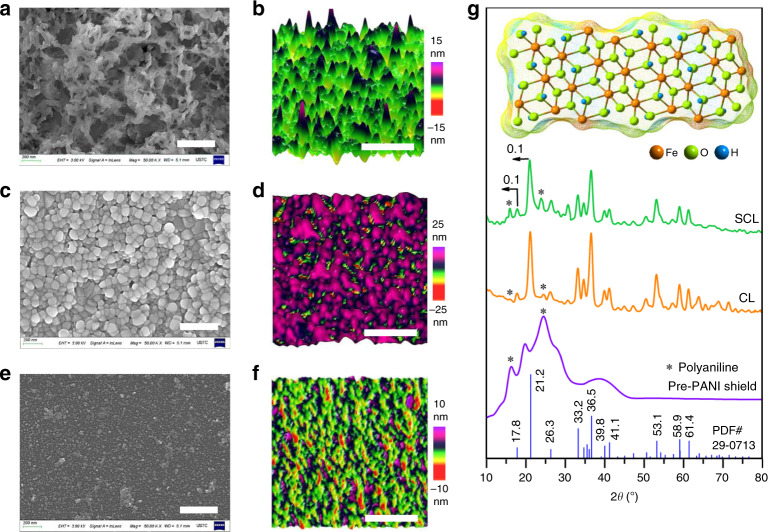
Physico-crystallinity analysis of the in-situ produced SCL. **a**, **c**, **e** SEM micrographs showing the grown pre-PANI nanostructures as a porous networked web at the Nafion-CEL surface, the produced Fe^+3^O(OH) catalyst nanoparticles and the post-PANI shield at the catalyst, respectively. **b**, **d**, **f** The AFM images in tapping mode at the three consecutive steps as **a**, **c**, **e** visualising the topographic features such as surface pits in the pre-PANI web, produced Fe^+3^O(OH) catalyst nanoparticles within the pits and the porous post-PANI shield. **g** Standard indexed peaks of the PDF#29-0713 card number for goethite Fe^+3^O(OH) material, XRD spectra of the pre-PANI shield, catalytic layer (CL) at pre-PANI and the shielded catalytic layer under post-PANI shield (SCL). Inset indicating a fully interconnected structure of goethite Fe^+3^O(OH) material. The scale bars in **a**, **c**, **e** correspond to 400 nm, whereas the scale bars in **b**, **d**, **f** are equal to 200 nm.

Four chemical reactions (Supplementary Fig. 1) govern in-situ synthesis and the shielding of the goethite catalyst at the Nafion-CEL surface. Beginning from the ANI seeds, which embed at the sulfonate groups of water swelled top surface of Nafion-CEL (Nafion—SO_3_^–^_···_^+^H_3_N—ANI, Stage 1 in Supplementary Fig. 1) were evaluated using CNS (Carbon, Nitrogen and Sulphur) elemental combustion analyser. The CNS analysis of three ANI seeded Nafion-CEL samples confirmed that almost 18 wt% of N (^+^H_3_N—ANI) is electrostatically entrenched with S (Nafion—SO_3_^–^) as ANI seeds (Supplementary Table 1). The seeds initiate hetero-nucleation to grow PANI nanostructures in such a way as to produce a porous web (pre-PANI shield, SEM in Fig. 2a) at the CEL surface. The pre-PANI shield contains nanosized surface pits (AFM in Fig. 2b) to grasp the FeCl_3_ solution and enable a very uniform catalyst formation. SEM and AFM images (Fig. 2c, d) show the in-situ produced Fe^+3^O(OH) catalyst nanoparticles of diameter 10–50 nm (details for the intermediate steps in catalyst synthesis are provided in Supplementary Fig. 1, Stage 3). The uniform distribution of the catalyst nanoparticles develops a catalytic layer (CL) at the pre-PANI shield. The in-situ growth of subsequent post-PANI shield covers the catalyst nanoparticles, as shown in Fig. 2e. The pre- and post-PANI shields beside the Fe^+3^O(OH) catalyst jointly develop a shielded catalytic layer (SCL) at the CEL surface. The AFM topographic image indicates pores of several nanometre depth in the SCL (Fig. 2f, following the colour bar), which are highly favourable for the infusion of dilute QPPO solution into the SCL to develop an engrained anion exchange polymer surface layer (AEL, discussed in details at a later stage).

The in-situ synthesis and successful shielding of the goethite Fe^+3^O(OH) catalyst were further confirmed with three times repeated XRD analysis at each synthesis step. Beginning from the XRD spectrum of pristine Nafion-CEL (Supplementary Fig. 2), which exhibits two wide XRD bands at 2*θ* degrees of 16.7 and 38.8, represent the Nafion material^18^. XRD spectrum of the pre-PANI shield (Fig. 2g) indicates the appearance of two additional XRD at 2*θ* degrees of 19.6 (benzenoid group) and 24.3 (quinonoid group), which specify the grown pre-PANI in electronically conductive emeraldine salt state (four-probe conductivity: 69.8 ± 0.9 μS cm^−1^). The appearance of distinguishably sharp XRD peaks, desertion of the peaks of Nafion material and diminishing of the XRD peaks of pre-PANI (marked with stars in Fig. 2g) jointly confirm the in-situ synthesis and uniform spreading of the crystalline nanoparticles at the pre-PANI shield (visible in SEM and AFM analysis, Fig. 2c, d, respectively). Careful XRD analysis of the CL with a materials data analysis software MDI Jade confirmed the matching standard card number PDF#29-0713 of goethite Fe^+3^O(OH) material (Fig. 2g) and Pbnm(62) space group. Wherein, perfect matching of the 10 indexed sharp diffractions at 2*θ* degrees of 17.8, 21.2, 26.3, 33.2, 36.5, 39.8, 41.1, 53.1, 58.9 and 61.4 confirm the successful formation of the crystalline goethite Fe^+3^O(OH) material at the pre-PANI shield. Same standard card number (PDF#29-0713) and diffraction angles in SCL (except a slight shift of the peaks at 2*θ* degrees of 17.8 and 21.2 towards the smaller angle, Fig. 2g) exhibit negligible distortion in crystallinity of the goethite material. However, restoration of the characteristic XRD peaks of PANI (marked with stars in SCL, Fig. 2g) exhibits the successful formation of the post-PANI shield. The CrystalMaker® Version 10.2.2 confirmed the synthesised goethite Fe^+3^O(OH) material, a fully interconnected orthorhombic stable structure (Inset of Fig. 2g and cif file in Supplementary Data 2)^19,20^. Briefly, the CNS combustion analysis, SEM, AFM and XRD analysis jointly confirm the successful formation of electronically conductive pre- and post-PANI shields beside the fully interconnected goethite Fe^+3^O(OH) material. Besides, analysis of the FTIR-ATR spectra of the pre-PANI shield, catalytic layer (CL) and shielded catalytic layer (SCL) compared with the Nafion-CEL are showing extra bands at 1591 and 1503 cm^–1^. This indicates the presence of PANI in its emeraldine salt state^21^. Moreover, the disappearance of all the peaks related to the Nafion and shielded catalytic junction (SCJ) and the appearance of new peaks of QPPO is demonstrating successful fabrication of the SCBMs (Supplementary Fig. 4).

### Excellent catalytic activity of goethite Fe^+3^O(OH) catalyst

**Fig. 3:**
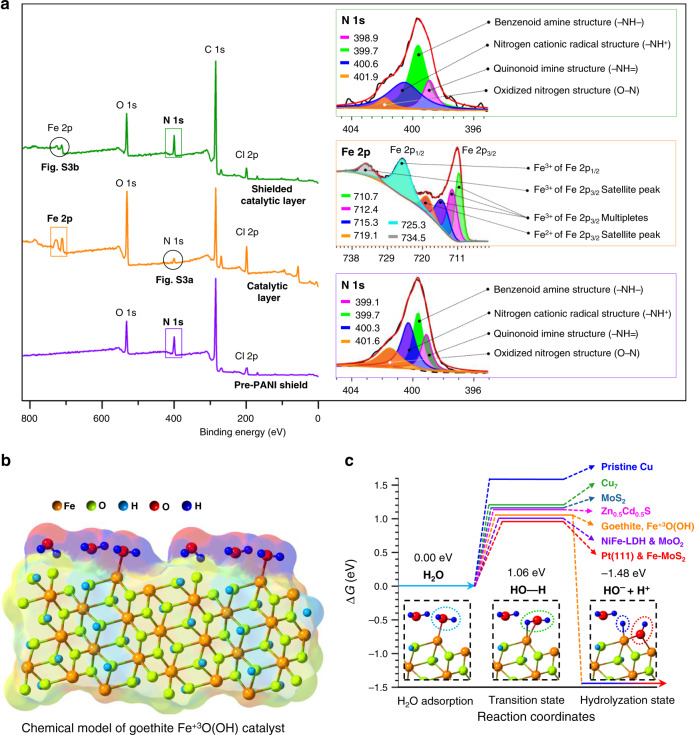
Evidence for in-situ synthesis, polyaniline shielding and efficient catalytic activity of the goethite Fe^+3^O(OH) catalyst. **a** The XPS survey spectra showing sharp characteristic peaks of N 1s and Cl 2p, whereas almost the same positioning of the deconvoluted peaks in the core-level XPS spectra of N 1s in pre-PANI shield (bottom Inset) and post-PANI shield (top Inset) are indicators of the electronically conductive doped PANI shields due to the presence of both the quinonoid imine (−NH**=**) and benzenoid amine (−NH**−**) structures. Moreover, the presence of nitrogen cationic radical structure (−NH^+^) and oxidised nitrogen structure (O**−**N) indicate electronically conductive and electrostatically bonded PANI shields (also confirmed in four-probe conductivity, FTIR-ATR and zeta-potential measurements). The sharp XPS peaks of Fe 2p in both the XPS survey spectra of CL and SCL, laying in the range of 705–740 eV along with deconvolved six peaks in the core-level XPS spectra (intermediate Inset) indicating the presence of Fe^+3^O(OH). **b** The basic chemical model and **c** the DFT-calculated Gibbs free energies confirming a sufficiently lower activation energy barrier of goethite Fe^+3^O(OH) catalyst (1.06 eV) than several famous catalysts. The energy for direct water dissociation without the use of any catalyst is ~5.15 eV per H–OH bond as shown in Supplementary Fig. 18a^36^. The catalysis activity of the goethite Fe^+3^O(OH) junctional catalyst is comparable to the several well-known non-junctional catalysts which have never been tested in shielded or non-shielded bipolar membranes.

Besides the detailed microscopy and crystallographic analysis, XPS of the pre-PANI shield, CL and SCL also confirm the in-situ synthesis and successful shielding of Fe^+3^O(OH) with electronically conductive polyaniline. The XPS survey spectrum of the pre-PANI shield (Fig. 3a) shows sharp characteristic peaks of N 1s ~400 eV and Cl 2p ~199 eV which are indicators of the successful growth of the doped pre-PANI shield. The deconvolution of the core-level XPS spectra of N 1s in the pre-PANI shield, which shows four peaks for quinonoid imine structure (―N=) at ~399.1 eV, benzenoid amine structure (―NH―) at ~399.7 eV, nitrogen cationic radical structure (―NH^+^) at ~400.3 eV and oxidised nitrogen structure (O―N) at ~401.6 eV, further confirm electronically conductive emeraldine salt state of the pre-PANI shield^22^. Notably, the protonated nitrogen (N^+^) peaks at binding energies higher than 400 eV attribute to the association of doping anion (Cl^–^, in this case) with the N^+^ and predicts strong electronic conduction characteristics of the pre-PANI shield^23,24^. Almost the same positionings of the four deconvoluted peaks in a core-level N 1s XPS spectrum of the post-PANI shield in SCL (Fig. 3a) was also observed, which confirm the in-situ formation and electronic conduction characteristics of the post-PANI shield as well. The presence of a sharp peak of the oxidised nitrogen structure (O−N) in the pre-PANI shield and a decrease in Zeta-potential from −0.952 mV for Nafion-CEL to −0.340 mV for Nafion-PANI indicates neutralisation/electrostatic binding between the negative sulfonate groups of the Nafion with the positive amine groups of PANI (Nafion—SO_3_^–^_···_^+^H_3_N—ANI).

Moreover, the increase in the asymmetry (variant potential distribution at the membrane surface) from −4.424 eV for Nafion-CEL to −1.353 eV for Nafion-PANI (Supplementary Table 2) predicts sporadic charge distribution, which is possibly due to the grown positively charged PANI nanostructures (Fig. 2a, b) at negatively charged Nafion-CEL surface. The appearance of new peaks of Fe 2p in the XPS survey spectra of CL and SCL (Fig. 3a) confirm the successful synthesis of the catalyst at pre- and under the post-PANI shields. Although, suppression of the Fe 2p peaks in the XPS survey spectrum of SCL (Fig. 3a) is observed, which is due to the post-PANI shield at the catalyst nanoparticles. However, the same binding energies in the XPS survey (705–740 eV) and core-level spectra of Fe 2p (around 711, 712, 715, 719, 725, and 734 eV, the intermediate Inset in Fig. 3a for CL and Supplementary Fig. 3a for SCL) confirm the presence of goethite Fe^+3^O(OH) catalyst in both the CL and SCL^25^. Thus, the analysis of the XPS survey and core-level spectra along with the XRD analysis jointly confirm the in-situ synthesis and successful shielding of the goethite Fe^+3^O(OH) catalyst layer within electronically conductive pre- and post-PANI shields.

After a very careful and detailed physico-crystallo-chemical analysis which confirm the in-situ synthesis and shielding of the goethite Fe^+3^O(OH) nanoparticles within the two electronically conductive polyaniline shields, we have performed DFT calculations to qualitatively understand the superior activity of goethite catalyst (find details in Supplementary Information and Supplementary Table 7). Although the entire process in the experiment is extremely complicated, water dissociation occurring at the catalyst surface is likely the rate-limiting step. Figure 3b shows a simplified model of the goethite catalyst surface containing adsorbed water molecules at the Fe^+3^ sites. Water adsorption and dissociation are both found at the terminated Fe^+3^ site and the corresponding Gibbs free energies are calculated for H_2_O adsorption (Δ*G*: 0.00 eV), the transition (Δ*G*: 1.06 eV) and the hydrolyzation (Δ*G*: −1.48 eV) states, respectively. Assuming zero effect of the externally applied reverse biased electric field (same as in literature and the DFT calculations in this work, all cif files that are used in the DFT calculations are provided as Supplementary Data 1)^26–32^, we have also calculated the relative energies to release the produced H^+^ (as H_3_O^+^) and OH^−^ from the catalyst surface (Δ*G*_release_: 5.06 eV, Supplementary Fig. 18b). However, the role of the electric field (second Wein effect) is always considered beneficial for WD as well as in releasing H^+^ (as H_3_O^+^) and OH^−^ from the catalyst surface and the junction^33–35^. Because the literature generally gives a comparison of the energies at the transition state by considering this as a fundamental energy barrier that is necessary to lower using various catalysts^26–33^. Therefore, we have compared the DFT-calculated energy barrier of the goethite Fe^+3^O(OH) catalyst at the transition state with the literature (Fig. 3c). The DFT calculations confirm that the goethite catalyst lowers the activation energy barrier of water dissociation from 5.15 eV (in water and without WD catalyst)^36^ to only 1.06 eV per OH–H bond at the transition state. Although the barrier lowering is not as significant as with Pt(111), Fe-MoS_2_, NiFe-LDH, MoO_2_ and other famous catalysts, the catalytic activity of the goethite is sufficiently better than several pristine catalytic materials such as Zn_0.5_Cd_0.5_S, Cu_7_, MoS_2_ and pristine copper^26–32^, which is certainly a great achievement. Most importantly, successful first-time use of the goethite Fe^+3^O(OH) catalyst in bipolar membranes for achieving energy-efficient water dissociation, indicates the significance of the present research work and importance of the produced catalyst in both the scientific and applied research.

### Deeper insight proves fast water dissociation in SCBMs

**Fig. 4:**
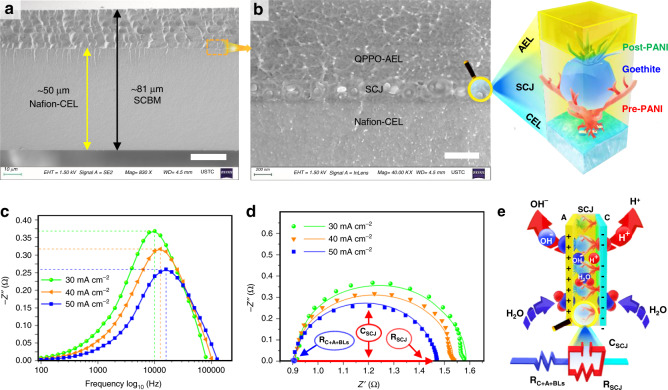
Physico-electrochemical analysis of the SCBMs. **a** SEM cross-sectional image showing a unified SCBM comprising ~50-µm-thick Nafion-CEL, ~31-µm-thick QPPO-AEL, and a clear junction. The scale bar corresponds to 20 μm. **b** A magnified SEM cross-sectional image and adjacent illustration exhibiting shielded catalyst nanoparticles at the C/AEL interface. The scale bar is equal to 400 nm. **c** The EIS Bode curves indicating their shift towards lower impedance and higher frequency values, which preliminary predict a decrease in water dissociation reaction resistance and improved junction hydration at larger current densities. **d** The EIS Nyquist plots, where shortened diameters of the semicircles predicting decreased charge transfer or water dissociation resistance at increased current densities^6^. The measured EIS data (symbols) was simulated with an EEC model (solid lines) to interpret the exact response of the membrane and SCJ. **e** The illustration representing the membrane and a best-suited Randles circuit model (fitting accuracy >97% and *χ*^2^ value <10^−4^), R_C+A+BLs_(C_SCJ_R_SCJ_). Here, the resistor R_C+A+BLs_, the capacitor C_SCJ_ and the resistor R_SCJ_ representing the cumulative CEL-AEL-BLs resistance, the junction capacitance and junction resistance^7^.

The infusion of QPPO solution within the pores of post-PANI shield followed by thermal solidification develop an engrained AEL at ionically bonded SCL with the Nafion-CEL. The resulting membranes of an almost 81 µm thickness composed of ~50-µm-thick Nafion-CEL, a relatively thinner (~31 µm) QPPO-AEL and a distinctive interface (Fig. 4a). Comparatively thinner AEL is favourable to overcome the slower permeation of OH^−^ than proton transportation in CEL^37^. A zoomed SEM cross-sectional view (Fig. 4b) and adjoining illustration provide more details about the interface such as the pre-PANI shield at the CEL surface, catalyst nanoparticles, the post-PANI shield around the particles and engrained AEL. Thus, the microscopy analysis confirms the formation of a unified SCBM containing in-situ produced and uniformly distributed goethite Fe^+3^O(OH) catalyst nanoparticles, protected between two electronically conductive PANI shields (as confirmed with XRD, FTIR-ATR and XPS analysis). Besides the detailed physico-crystallo-chemical analysis, the electrochemical response of the SCBMs is preliminarily assessed using electrochemical impedance spectroscopy (EIS) analysis at AUTOLAB workstation using two-compartment glass cell (Supplementary Fig. 6, also find the testing details in Supplementary Information). Besides, the electrochemical characteristics of the SCJ such as electro-ionically neutral junction region (*λ*), junction capacitance and water dissociation or charge transfer resistance, were also quantitatively calculated by an electrical equivalent circuit (EEC) modelling and simulation of the EIS data at ZSimpWin^38^.

**Fig. 5:**
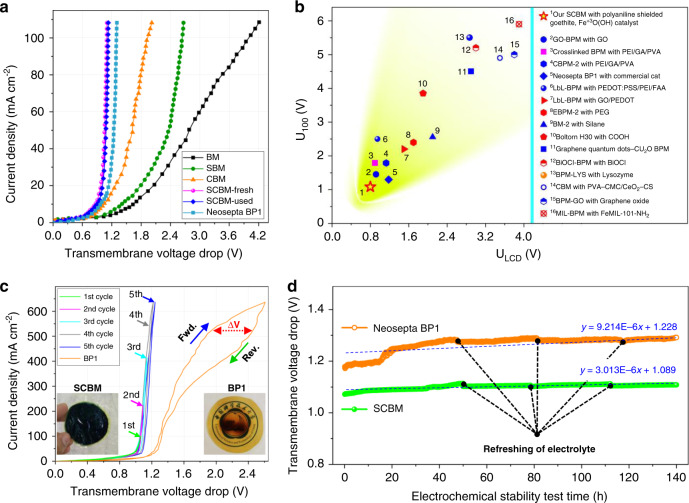
Galvanostatic polarisation analysis confirm fast and stable water dissociation. **a** The *I*–*V* curves up to 109 mA cm^−2^ current density for the bipolar membranes without catalyst (BM), containing only the PANI shields (SBM), containing only the ex-situ synthesised and spray-deposited catalyst (CBM), fresh SCBM, thoroughly used SCBM, and a fresh commercial Neosepta BP1. The polarisation curves indicating the requirement of a very small WD voltage at the LCD and 100 mA cm^−2^ current densities with the SCBMs (*U*_LCD_: 0.8 V and *U*_100_: 1.1 V) than the BP1 (*U*_LCD_: 1.1 V and *U*_100_: 1.3 V). **b** Comparative electrochemical characteristics showing very low *U*_LCD_ and *U*_100_ voltages for the SCBMs (presented as a star at the lower right corner) than the previously reported CBMs. **c** The electrochemical stability test of the SCBMs and BP1 membranes during fast water dissociation. Initial four *I*–*V* cycles (1st–4th) were recorded for a fresh SCBM at loading–unloading–reloading current densities between 0 to 102, 223, 414, and 637 mA cm^−2^ (within the testing limit), whereas the 5th cycle is for the same SCBM sample after a thorough use in EIS, *I*–*V* and SCBM-ED tests. A highly reversible trend in the *I*–*V* cycles is indicative of excellent electrochemical stability and membrane durability during ultrafast water dissociation at high operating current densities (right Inset, an undamaged SCBM). In contrast, a fresh BP1 scorched (left Inset, a symptom of the junction dehydration) during the stability test and could not behave reversibly after 300 mA cm^−2^ (large passive potential difference, ∆*V*). **d** Long-time electro-stability (almost 6 days, 140 h) of the membranes during water dissociation at 100 mA cm^−2^ (within the testing limitation of AUTOLAB electrochemical workstation, PGSTAT 302N, Metrohm, Netherland). Linear fitting with blue lines (*y* = *m*x + *c* where *m* and *c* are slope and y-intercept, respectively) on the stability data indicates smaller transmembrane potential drop (*c* = 1.089 V) and minor decline in water dissociation rate of SCBMs (*m* = 3.013 µV min^−1^) compared with BP1 (*c* = 1.228 V, and *m* = 9.214 µV min^−1^). The black dotted regions indicating sudden potential drops in the experimental data due to the refreshing of the electrolytes in the testing cell.

The galvanostatic EIS analysis is performed at three current densities such as 30, 40 and 50 mA cm^−2^ for both the SCBMs and the commercial FumaTech FBM membranes (for comparison). The value of *λ* is calculated using three sets of the frequencies and corresponding imaginary impedance values at the apex point of each Bode curve of the SCBMs (Fig. 4a) and FBMs (Supplementary Fig. 7a) using a simplified mathematical expression (Eq. 1 in Methods section)^39^. During repeated EIS analysis, we found a linear relationship of the *λ* in SCBMs (λ_SCJ_: 83.4 < 90.6 < 93.1 nm) and *λ* in FBMs (λ_FBJ_: 10.4 < 11.7 < 13.6 nm) with the applied current densities (30 < 40 < 50 mA cm^−2^). Herein, almost 89.1 nm junction region is electro-ionically neutral from ~300-nm-thick shielded catalytic junction (visible in the SEM cross-sectional image, Fig. 4b) containing PANI shields, catalyst and penetrated QPPO (see the distribution of bromine, a representative element of QPPO, in Supplementary Fig. 5). However, ~11.9 nm is the electro-ionically neutral catalytic junction in FumaTech FBM (Supplementary Table 4). A similar increasing trend in the junction capacitance (*C*_SCJ_: 18.7 < 21.1 < 21.9 µF cm^2^) and (*C*_FBJ_: 4.8 < 4.9 < 5.2 µF cm^2^) at increased current densities was obtained from the EEC modelling and the data simulation (fitting accuracy: 97% and *χ*^2^ value <10^−4^). The increase in *λ* (thickness of the electro-neutral junction region) and *C* (junction capacitance) with the increased current densities are likely due to a voltage-dependent change in ion concentrations during a WD process inside the membrane junction. However, comparatively larger *λ*_SCJ_ (89.1 nm) and *C*_SCJ_ (20.6 µF cm^2^) predict spacious junction of the SCBMs than FumaTech FBM (*λ*_FBJ_: 11.9 nm and *C*_FBJ_: 4.9 µF cm^2^). This junctional characteristic is highly beneficial for uptaking large feed water to avoid membrane dehydration and enabling fast water dissociation (Fig. 5c and Supplementary Fig. 11b)^40^. The electro-ionically insulated large region is also possible due to the porous morphology of the shielded catalytic junction (smaller Q-n_PCI_: 0.603, Supplementary Table 5). The small value of cumulative resistance of the SCBMs (*R*_C+A+BLs_: 0.906 ± 0.006 Ω cm^2^) compared with the FumaTech FBM (*R*_C+A+BLs_: 1.41 ± 0.01 Ω cm^2^) is also beneficial for enabling the quick supply of feed water to the junction and withdrawal of H^+^/OH^−^ ions across the C/AEL of SCBMs. Another most important junctional characteristic is the water dissociation reaction resistance (*R*_SCJ_) and its significant decrease with increase in current densities such as 0.679 Ω cm^2^ at 30 mA cm^−2^ to 0.627 Ω cm^2^ at 40 mA cm^−2^ (7.66% decrease) and 0.549 Ω cm^2^ at 50 mA cm^−2^ (12.44% decrease) dictates an enhanced rate of water dissociation in SCBMs at high current densities (second Wein effect)^33–35^. Moreover, a comparatively smallest resistance (*R*_CJI_: 1.961 Ω cm^2^, *R*_PCI_: 1.293 Ω cm^2^ and *R*_JAI_: 1.779 Ω cm^2^) and largest admittance (Q-Y_CJI_: 0.002 S cm^−2^ s^*n*^, Q-Y_PCI_: 0.043 S cm^−2^ s^*n*^ and Q-Y_JAI_: 1.592E-4 S cm^−2^ s^*n*^) of the polyaniline-catalysts-interface (PCI) at both the 1 and 3 mA cm^−2^ predict fast ionic transportation and WD within the junction due to the abundance of PANI and the catalyst (Fig. 4b, Supplementary Fig. 8 and Supplementary Table 5). These results are theoretically in accordance with Faraday’s law of charge conduction (i.e., more the charge carriers, less the charge transfer resistance)^7,41^. Briefly, the EIS analysis preliminary validates the electrochemical excellence of the SCBMs compared with the commercial FumaTech FBMs such as large water uptake, fast water dissociation, and quick withdrawal of the produced H^+^/OH^−^ ions at increased current densities.

### Energy-efficient and stable water dissociation performance

Besides, the detailed physico-crystallo-electrochemical analysis and the DFT calculations of the SCBMs, individual role of the PANI shields, the goethite Fe^+3^O(OH) catalyst and their in-situ produced composite on WD performance are also evaluated by galvanostatic polarisation analysis. For this purpose, various bipolar membranes are fabricated without catalyst (BMs), with PANI shields (SBMs), with the ex-situ synthesised catalyst (CBMs) and the membranes containing in-situ PANI shielded catalyst (SCBMs). The galvanostatic polarisation (direct current density *vs*. transmembrane voltage drops) as *I*–*V* curves are recorded up to 109 mA cm^−2^ current density (Fig. 5a) using a four-compartment glass cell as shown in Supplementary Fig. 9. The SBMs compared with the BMs not only offer significantly decreased voltage drop but also significantly enhanced WD performance (curve turns to vertical) at the same current densities. However, the deviation of the curve from the vertical trend (Fig. 5a) and small blisters, which appeared in BMs (Supplementary Fig. 14f) during the *I*–*V* test is exhibiting the disadvantage of the spray-deposited ex-situ synthesised catalysts. Among all the membranes, the SCBMs enabled the lowest reverse bias electric potential drop (0.8 V at limiting current density or LCD) which is much smaller than the voltage required by the benchmark Neosepta BP1 (1.1 V at LCD, Fig. 5a), FumaTech FBM, and several reported CBMs (Fig. 5b and Supplementary Table 6). Moreover, the transmembrane voltage drops at 100 mA cm^−2^ (typical value of industrially applied current density for optimum water dissociation performance) is also relatively much lesser (below 1.1 V) than commercial and numerous reported CBMs (Supplementary Table 6). The vertical trend in *I*–*V* curves after 40 mA cm^−2^ illustrates an electric field enhanced steady WD performance with the SCBMs due to electronically conductive PANI shields (the difference in vertical trend is more visible in the *I*–*V* curves of SBMs with conductive shields of PANI emeraldine salt and NSBMs with non-conductive shields of PANI emeraldine base, Supplementary Fig. 13c)^7,42^. This trend is also evident in the EIS analysis as an increase in the rate of water dissociation which is due to a decrease in water dissociation reaction resistance at comparably increased current densities (Supplementary Tables 3 and 5)^7^. The water dissociation measurements are usually affected at low current density due to co-ion leakage across the bipolar membranes, leading to a parasitic or charge-compensating current. The *I*–*V* curves at a low current density (2 mA cm^−2^, Supplementary Fig. 12a) indicate varied transmembrane voltage drop (linearly related to the parasitic current) for all the fabricated and commercial bipolar membranes in this work. Herein, the voltage drop (parasitic current) in the representative SCBM-fresh and SCBM-used membranes is almost comparable with the FumaTech FBM and much lower than the Neosepta BP1 at the same current density. At high current densities this leakage/parasitic current becomes negligible in the SCBMs compared with all other bipolar membranes, as observable from the negligible voltage drop with the increase in current density (Supplementary Fig. 12b). Briefly, a simultaneously smaller voltage for the WD at LCD (*U*_LCD_: 0.8 V) and steady WD at 100 mA cm^−2^ current density (*U*_100_: 1.1 V) signify the beneficial use of the shielded goethite catalyst and place the fabricated SCBMs at a distinguished position in a comparison of various CBMs, as represented with a star at lower right corner in Fig. 5b.

In addition, cyclic loading, unloading and reloading of the current densities from 0 to 102, 223, 414 and 637 mA cm^−2^ as 1st to 4th cycles in Fig. 5c indicate a negligible passive transmembrane potential drop and exhibit a highly reversible trend in the *I*–*V* curves of fresh SCBMs. Herein, 637 mA cm^−2^ is taken as a cut-off point for the cyclic stability test because of an unusual increase in the temperature of the test solution (approaching 60 ^o^C at a higher current density). The SCBMs exhibit a good long-time electro-stability (rate of voltage drop: 12 μV min^−1^ within the testing limitations) at 637 mA cm^−2^ WD current density and perfect reversibility (negligible passive potential drop) as shown in Supplementary Fig. 10 (Inset). Afterward, a thoroughly used SCBM during EIS, *I*–*V* and SCBM-ED processes re-exhibited excellent stability (the highly reversible trend in the *I*–*V* curve, 5th cycle in Fig. 5c). Near-vertical and highly reversible *I*–*V* curves demonstrate energy-efficient and stable WD performance of the SCBMs, which is possible due to several factors. The factors include fully interconnected characteristic of the novel goethite Fe^+3^O(OH) catalyst, catalyst shielding within electronically conductive polyaniline (Fig. 3a and Supplementary Fig. 13) which allows spatial separation of the catalyst from the acidic Nafion-CEL surface, the spatial position of the catalyst inside the polyaniline shielded extended junction (~300 nm, which can regulate the electric field distribution inside the SCL)^43^, and a larger active surface area of the nanoparticles because of increased ionic access inside the junction. Moreover, no signs of physical deterioration such as blistering or burning during EIS, *I*–*V*, SCBM-ED and stability tests (right Inset of Fig. 5c) ensure excellent electrochemical durability of the fabricated SCBMs.

In contrast, the commercial Neosepta® BP1 encountered a large passive transmembrane potential drop (Δ*V*) during the cyclic water dissociation stability test even after 300 mA cm^−2^ (Fig. 5c) and badly damaged (scorched, left Inset in Fig. 5c). Similarly, the commercial FumaTech FBM also showed worse cyclic electro-stability (very large passive transmembrane potential drop in each cycle, Supplementary Fig. 11b) and even could not behave reversibly and failed structurally after 450 mA cm^−2^ (Supplementary Fig. 11b, Inset). The junction dehydration issue in BP1 and FBM probably happens due to insufficient junction region and resistive fibrous support, which imbalance the rate of water uptake and withdrawal of the reaction products (H^+^/OH^−^), leading to their physico-chemical failure^4^. Besides a very low transmembrane voltage drop and high cyclic stability, SCBMs also exhibited excellent electro-stability during long-time water dissociation for almost 6 days (140 h, Fig. 5d) at 100 mA cm^−2^. Only 3.013 µV min^−1^ passive potential drop occurred in SCBM, which is almost 3.1× lesser than the passive potential drop in Neosepta BP1 (9.214 µV min^−1^, Fig. 5d). Moreover, the long-time electro-stability of the SCBMs is also extraordinarily better than FumaTech FBM bipolar membrane (Supplementary Fig. 11c). Thus, the SCBMs which enable water-dissociation at very low voltage and exhibit excellent electro-stability (negligible passive potential drop) can be confidently applied in advanced water dissociation processes at sufficiently large current densities (637 mA cm^−2^, within the testing limit, Fig. 5c and Supplementary Fig. 10).

### Fast in-situ acid–base generations with SCBMs in the ED process

**Fig. 6:**
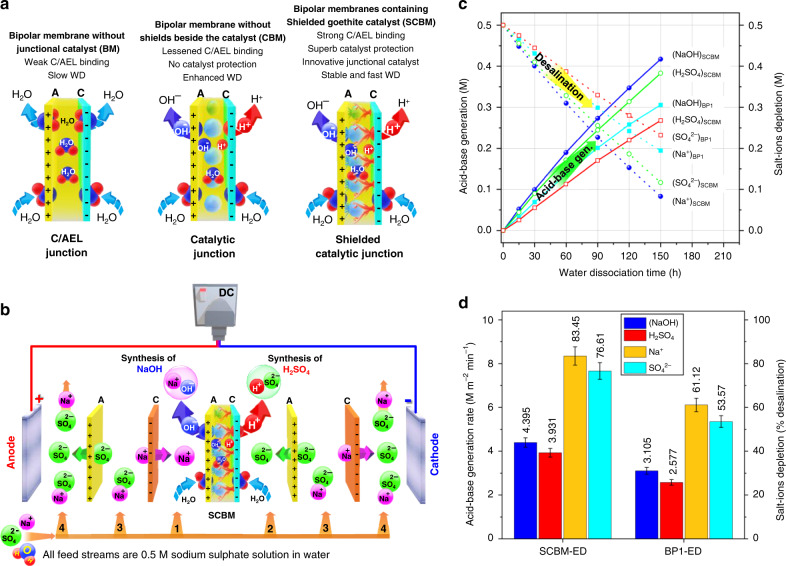
SCBMs-ED approves fast in-situ acid–base generations and saline-water desalination. **a** Simplistic models exhibiting the excellent role of junctional catalyst and polyaniline shields. **b** The SCBMs-integrated electrodialysis single-cell, comprising SCBMs at the centre, surrounded by two sets of commercial anion–cation exchange membranes (Neosepta® AMX and CMX, denoted here as A and C, respectively). The arrangement of cell plates develops four types of compartments: 1-base, 2-acid, 3-auxiliary and 4-electrode rinsing solution, sequentially from the centre of the cell towards the electrodes. Notably, AEL surface of the SCBMs (7.07 cm^2^ effective area) is orientated facing the anode compartment. Four batches of 200 ml 0.5 M Na_2_SO_4_ salt solution were separately supplied and continuously circulated within each type of the compartment. The water continuously diffuses inside the SCJ, wherein the catalyst dissociates the feedwater and C/AEL withdraw the resulting H^+^/OH^−^ ions under applied reverse-bias DC potential for an in-situ acid-base generation. **c** The acid-base generation and saline-water desalination performance with ED time and **d** the acid–base generation rate and percentage saline-water desalination (within data variation of ±5% s.d.) indicating the significantly better performance of the SCBMs than the commercial BP1 membranes.

The analysis of galvanostatic EIS and *I*–*V* curves anticipate an ultrafast influx of feed water, quick water dissociation within the SCJ and rapid out-migration of H^+^/OH^−^ ions across the C/AEL pair due to the small values of membrane and water dissociation reaction resistances. Based on the physico-electrochemical analysis (Figs. 2–5, and the photographs in Supplementary Fig. 14), we have summarised the role of junctional catalyst and shields as simplistic models (Fig. 6a). Briefly, the bipolar membranes without any junctional catalyst although showed some binding of the C/AEL pair but required huge water dissociation voltage (slow water dissociation) due to WD catalysis at the membrane end-groups facing the junction. On the other hand, the use of catalyst sufficiently lowered the WD voltage (enhanced the WD performance) but weakened the C/AEL binding, resulting in quick membrane failure. However, the SCBMs containing the in-situ grown electronically conductive polyaniline shields and the goethite junctional catalyst not only lowered the water dissociation voltage but also lessened the catalyst loss and improved the C/AEL binding.

Besides just the evaluation of the water dissociation performance of the CBMs, we have also applied the fabricated SCBMs in electrodialysis cell (SCBMs-ED, Fig. 6b) and tested the rate of in-situ acid–base generations and saline-water desalination performance. Notably, the electrochemical performance in this work is based on a single-cell SCBMs-ED setup, where the AEL of SCBMs is orientated facing the anode. However, industrial CBM-ED setups employ stacks of such cells to achieve more substantial acid-base generation performance. Unlike the water electrolysis process which yields hydrogen and oxygen gasses as key products. A CBM polarises water within the catalytic junction under reverse bias DC potential and split

the water molecules into protons and hydroxide ions. The externally applied electric also contributes to removing the produced H+ and OH^−^ ions out from the catalytic junction^33–35^, toward the counter electrodes by permeating through the C/AEL pair and consume the available salt-anion (SO_4_^2−^) and salt-cation (Na^+^) to generate acid (H_2_SO_4_) and base (NaOH), respectively. Figure 6c shows linearly increased acid–base generations and a simultaneously decreased concentration of the salt-ions (saline-water desalination). Relatively much faster acid-base generations and the desalination performance were observed with the SCBMs than the commercial BP1 and the commercial FumaTech FBM bipolar membranes. Moreover, the depletion of ~84% Na^+^ and ~77% SO_4_^2−^ salt-ions within only 150 min and fast generation rate of H_2_SO_4_ (3.9 ± 0.19 M m^−2^ min^−1^) and NaOH (4.4 ± 0.21 M m^−2^ min^−1^) indicate extraordinary SCBMs-ED single-cell performance (Fig. 6d). In contrast, the ED performance of the commercial FumaTech FBM (~66% of Na^+^ and ~63% of SO_4_^2−^ salt-ions depletion along with only 3.0 ± 0.15 M m^−2^ min^−1^ of H_2_SO_4_ and 3.2 ± 0.16 M m^−2^ min^−1^ of NaOH) and commercial Neosepta® BP1 (~ 61% of Na^+^ and ~54% of SO_4_^2−^ salt-ions depletion along with only 2.6 ± 0.13 M m^−2^ min^−1^ of H_2_SO_4_ and 3.1 ± 0.16 M m^−2^ min^−1^ of NaOH) are deficient, as shown in Supplementary Fig. 15b and Fig. 6d, respectively. Moreover, the SCBMs-integrated ED process is showing high current efficiency (~99%) compared with the commercial FumaTech FBM (~83%) and Neosepta BP1 membranes (~71%) at the same operational conditions. Thus, the SCBMs are highly suitable to confidently apply in advanced sustainable technologies due to shallow DC-potential requirements for the water dissociation, extraordinary membrane durability, fast in-situ acid-base generations, and large current efficiency^44–46^.

## Discussion

Our results clearly confirm that the novel goethite Fe^+3^O(OH) catalyst with an activation energy barrier of only 1.06 eV (uncatalyzed energy barrier for WD is 5.15 eV) per OH–H bond is lower than many pristine catalytic materials, is significantly important for both the scientific research and industrial applications. Particularly, the conceivable simplistic in-situ synthesis, highly-interconnected stable structure, and low-cost are salient features to apply the goethite catalyst in CBMs. Besides, the proposed in-situ catalyst shielding with heterogeneously grown polyaniline is also significantly beneficial for not only protecting the catalysts but also fabricating the ever-desired durable bipolar membranes. The resulting SCBMs exhibited significant water uptake, quick start-up and energy-efficient water dissociation (*U*_LCD_: 0.8 V and *U*_100_: 1.1 V), small water-dissociation reaction resistance, and instant recovery of H^+^/OH^−^ ions. The SCBMs also showed excellent cyclic performance up to 637 mA cm^−2^ (within the testing limit) and long-time electro-stability for longer than 140 h at 100 mA cm^−2^, which is 3.1× better than commercial Neosepta® BP1 and extraordinarily better than commercial FumaTech FBM. The SCBMs in an electrodialysis single-cell also enabled ultrafast in-situ acid–base generations such as 3.9 ± 0.19 M m^−2^ min^−1^ of H_2_SO_4_ and 4.4 ± 0.21 M m^−2^ min^−1^ of NaOH at 100 mA cm^−2^, which are significantly faster than the Neosepta BP1 (2.6 ± 0.13 M m^−2^ min^−1^ of H_2_SO_4_ and 3.1 ± 0.16 M m^−2^ min^−1^ of NaOH) and FumaTech FBM (3.1 ± 0.15 M m^−2^ min^−1^ of H_2_SO_4_ and 3.2 ± 0.16 M m^−2^ min^−1^ of NaOH). Thus, the fabricated SCBMs containing polyaniline shielded goethite catalyst of excellent electrochemical performance and stability can confidently be applied for in-situ acid–base supplies at the same rate as their consumption in AP, HER, OER and CO_2_ reduction processes. Thus, the reported inexpensive novel goethite Fe^+3^O(OH) catalyst may be an appropriate alternative of several expensive catalysts. In a broader sense, the proposed facile aniline seeding, heterogeneous growth of polyaniline nanostructures, and the in-situ shielding strategy may be extended to chemically decorate the GO sheets, metal oxides, DNA templates, CNTs and polymers as well as electrochemically stabilise various catalysts for diverse applications in energy storage devices, biological sensors, electrocatalysis and membranes.

## Methods

### Materials

Aniline (99.5%) from Energy Chemicals China, and all other reagent grade chemicals including hydrochloric acid (36.46%), iron(III) chloride (FeCl_3_, anhydrous), sodium hydroxide (NaOH), sodium chloride (NaCl), sodium sulphate (Na_2_SO_4_) and methanol were purchased from Sinopharm Chemical Reagents Co. Ltd. China and used as received. Commercial perfluoro sulfonic Nafion® 212 membranes of thickness 50 µm were used as a cation exchange layer (Nafion-CEL). Quaternized poly(2,6-dimethyl-1,4-phenylene oxide) solution in methanol, kindly supplied by Hefei Chemjoy Co. Ltd. China was used for the formation of the anion exchange layer (QPPO-AEL). Two sets of Neosepta® CMX (cation exchange membranes) and Neosepta® AMX (anion exchange membranes) were purchased from Tokuyama Co., Japan and used as auxiliary membranes during the electrochemical bipolar membrane integrated electrodialysis (BM-ED) processes for analysis of water dissociation, acid–base generation and saline-water desalination. Neosepta® BP1 (Tokuyama Co., Japan) was used as a standard bipolar membrane for comparison purposes. Deionized (DI) water was used throughout the research work.

### Fabrication of shielded catalytic bipolar membranes

The SCBMs were fabricated in a two-flanged vertical glass cell by following the five sequential steps including four in-situ chemical reactions for the formation of a shielded catalytic layer (SCL) and fifth the physical infusion of anion exchange polymer solution into the SCL for AEL formation (Fig. 1a and Supplementary Fig. 1). While the SCBM fabrication, the chemical reactions such as aniline (ANI) seeding, growth of polyaniline (PANI) nanostructured web, the evolution of FeCl_3_ into Fe(OH)_3_, growth of post-PANI and physical infusion of QPPO solution within the pores of the shielded catalytic layer (SCL) were all performed in the upper half of the reaction cell holding an acidified Nafion-CEL (Supplementary Fig. 1).

Initially, the reaction cell was filled with 0.3 M aniline solution in 1 M HCl (25 ml) and the seeding mechanism was continued for 12 h. While ANI seeding, the amine functionalities of aniline monomer (H_2_N—ANI) accept protons from the sulfonic acid groups of the acidified Nafion layer (Nafion—SO_3_^–^ H^+^) and produce anilinium sulfonate salt (Nafion—SO_3_^–^_···_^+^H_3_N—ANI), which embed as ANI seeds within the water swelled top surface of the Nafion-CEL (Stage 1 of Supplementary Fig. 1 and Supplementary Table 1). After removing the ANI solution, the reaction cell was refilled with FeCl_3_ solution in DI water (0.3 M FeCl_3_, 25 ml) for 2 h, which nucleating from the ANI seeds initiates heterogeneous substrate-based polyaniline growth and produce electrostatically attached PANI nanostructured porous web (filled with FeCl_3_ solution) at the Nafion-CEL surface (Stage 2 of Supplementary Figs. 1 and 2a). Later, sodium hydroxide solution (1 M, 25 ml) was added into the FeCl_3_ solution for 1 h, which evolve the FeCl_3_ solution into Fe(OH)_3_ at intermediate Stage 3 of Supplementary Fig. 1. The reaction cell was gently cleaned with DI water and the Fe(OH)_3_ at pre-PANI containing Nafion-CEL was heated at 60 °C for 12 h to convert the Fe(OH)_3_ at pre-PANI web into goethite Fe^+3^O(OH) catalyst. The cell was refilled with aniline solution (0.3 M in 0.1 M HCl, 25 ml) for subsequent in-situ polymerisation reaction of 30 min. Herein, the HCl partially deteriorates the surface of Fe^+3^O(OH) nanoparticles and produces FeCl_3_ which polymerises ANI to protect the Fe^+3^O(OH) nanoparticles beneath a porous post-PANI shield (Stage 4 of Supplementary Figs. 1 and 2f). The pre- and post-PANI shields beneath and above the nanoparticles protect the catalyst to avoid its loss during electrochemical processes. Finally, 5% w/w of QPPO solution in methanol was poured at the thoroughly cleaned and dried shielded catalytic layer (SCL), which diffuse inside the pores of SCL to produce an almost 31-µm-thick anion exchange layer (Fig. 4a, b) and shielded catalytic junction (SCJ) at the C/AEL interface. All the steps for the formation of the SCL were performed at 13 ± 1 °C, whereas the AEL was formed in a drying oven at 40 °C. Finally, the unified bipolar membrane composing of the shielded catalytic junction (henceforth denoted as SCBMs) was removed from the glass cell and either put in a desiccator or immersed into the test solutions for further characterisation. Besides, the AEL was also formed at different intermittent steps such as at pristine Nafion-CEL to form blank bipolar membrane (BMs), at the in-situ grown emeraldine salt form of polyaniline on the Nafion-CEL (Nafion_PANI-ES) to form shielded bipolar membrane (SBMs), at the in-situ grown emeraldine base form of polyaniline on the Nafion-CEL (Nafion_PANI-EB) to form non-conductive shielded bipolar membranes (NSBMs), at the ex-situ synthesised and sprayed catalyst on the Nafion-CEL (Nafion_Catalyst) to form catalytic bipolar membrane (CBMs). Notably, polyaniline was converted into the base form (Nafion_PANI-EB) by immersing Nafion_PANI-ES in 1 M NH_3_ solution for 1 h via the de-doping process.

### Microscopy (SEM and AFM) analysis

The microscopy analysis at each sequential step such as the pre-PANI shield, the catalyst layer at the pre-PANI, post-PANI shield and the final SCBM (including elemental mapping) was performed using field-emission scanning electron microscope (FE-SEM, GeminiSEM 500, ZEISS, Germany) with energy dispersive spectroscopy detector (EDS, Silicon Drift Detector (SDD)-X-MaxN, OXFORD). The surface morphology at pre- and post-PANI shields and the catalyst layer was also investigated using an atomic force microscope in tapping mode (AFM, DI MultiMode V SPM, Veeco Instruments Inc. UK). For clearer interpretation, the resulting topographic height data was processed using NanoScope Analysis software 1.8 (Bruker Corporation, USA).

### X-ray diffraction analysis

The repeated XRD analysis at various synthesis steps such as Nafion-CEL, pre-PANI shield, catalyst layer and the shielded catalytic layer was performed at Rigaku X-ray diffractometer model TTR-III (Tokyo, Japan) with Cu Kα radiation over a range of 2*θ* degree of 10–80 at a scan rate of 10° min^−1^. The XRD spectra were carefully analysed with a materials data analysis software, MDI Jade.

### X-ray photoelectron spectroscopy analysis

The XPS analysis at various synthesis steps such as the pre-PANI shield, catalyst layer, and the shielded catalytic layer was performed at Thermo ESCALAB 250 with monochromatic Al Kα radiation source of hv 1486.6 eV, X-ray spot of 500 μm, and fixed transmission energy of the energy analyser is 30 eV. The XPS core-level spectra were carefully analysed with a peak fitting XPSPEAK software.

### Fourier transform infrared spectroscopy

Thermo Nicolet iS10 FTIR with SMART OMNI Transmission and SMART iTR (Thermo Fisher Scientific, USA) was used for the FTIR spectroscopy in attenuated total reflection mode (FTIR-ATR). Zinc selenium (ZnSe) and germanium (Ge) crystals were used as background medium and spectra of all the membranes were obtained in 650–4000 cm^–1^ wavenumber range.

### Zeta-potential measurements

An electro-kinetic analyser (SurPASS^TM^ 3 Eco instruments for routine solid surface analysis) was used to measure the zeta potential of the membrane samples at room temperature in 1 mM KCl solution at 5 pH.

### Density functional theory calculations

The water and polymer can contaminate the catalyst surface and makes the surface terminations very complex. Therefore, we have preferred to perform the DFT calculation via the XRD analysis of the catalytic layer (CL) and the shielded catalytic layer (SCL) at the Nafion-CEL surface (Fig. 2g). The XRD pattern is matched with goethite Fe^+3^O(OH) at a prominent characteristic crystal face (1 1 0). Therefore, we considered (1 1 0) crystal face as a major contributor to the catalysis reaction for Fe^+3^O(OH). We cleaved the Fe^+3^O(OH) crystal by (1 1 0) crystal face using CASTEP program in Material Studio package of Accelrys Inc.^47^ and decided our reaction path for the DFT calculations. Perdew-Burke-Ernzerhof (PBE) exchange-correlation functional of generalised gradient approximation (GGA) was employed with the ultrasoft pseudopotentials (USP). The number of the plane wave was determined by an energy cut-off of 380 eV. The structure optimisations were carried out in Brillouin zone which was sampled by a 1 × 1 × 1 k-points grid. The convergence tolerances for energy, maximum displacement, and maximum force were set to 2.0 × 10^−5^ eV per atom, 0.02 Å and 0.05 eV Å^−1^, respectively. The transition state was searched using complete linear synchronous transitions (LST) and quadratic synchronous transitions (QST) approaches. The periodic structure for the DFT calculations was made by a 9.16 Å × 10.36 Å × 8.41 Å supercell with a vacuum region of 10 Å between the slabs along the *z*-axis.

### Electrochemical impedance spectroscopy analysis of SCBMs

The galvanostatic EIS analysis was performed to calculate the junction thickness, junction capacitance and water dissociation reaction resistance. The EIS testing was performed in a two-compartment electrochemical glass cell based on the standard Kelvin four-point method, as schematically shown in Supplementary Fig. 6. Both the compartments were separated with the SCBM sample of 10.18 cm^2^ active area and filled with a 0.5-M NaCl solution. The solution in both the compartments was continuously mixed and circulated with peristaltic pumps at a flow rate of 30 mL min^−1^ to minimise the concentration polarisation effects. The cell was equipped with platinum sheets (5 × 10 mm) as working (W) and counter (C) electrodes, whereas reference (R) and sense (S) were Ag/AgCl electrodes, located at almost one millimetre apart on both sides of the bipolar membrane surface through Haber-Luggin capillaries. The EIS data were recorded using AUTOLAB electrochemical workstation (PGSTAT 302N, Metrohm, Netherland) in galvanostatic mode, controlled by a computer with Nova 2.1.2 software. The EIS testing was performed under high stability mode, where each experimental data point was taken as an average of 10 acquired points and presented as typical Bode and Nyquist plots. The frequency (*ν*_max_) and imaginary impedance values (*−Z″*_max_) at the apex point of the Bode curves (Fig. 4c) were used to calculate the junction thickness (*λ*) using a derived Eq. 1^39,48^.

1 $$\lambda = \varepsilon _{\mathrm{0}}\varepsilon _{\mathrm{r}}A_{ef} \times 4\pi \left( {-{Z}^{\prime\prime}}_{\mathrm{max}} \right)\nu _{{\mathrm{max}}} \times 10^{ - 7}$$

where *ε*_0_ is the vacuum permittivity with a value of 8.854, *ε*_r_ is the relative dielectric constant (typical value is 20 for polymeric membrane)^38^, and *A*_*ef*_ is the effective contact area (For the SCBMs: 10.18 cm^2^ and for the FBM: 3.2  cm^2^) which is equivalent to ten times of the actual effective area for bipolar membranes^17,49^. Furthermore, the EIS complex impedance data, presented as Nyquist plots in Fig. 4d is simulated using electrochemical analysis software (ZSimpWin, P.A.R. Inc. USA) and assessed several characteristics of the SCBMs^40,50^. The well-matched modified Randles equivalent electrical circuit (EEC) model, R_C+A+BLs_(C_SCJ_R_SCJ_) was fitted and simulated at the experimental EIS Nyquist data which exhibited 97% of the fitting accuracy (Chi sq, *χ*^2^ < 10^−4^). The resulting values of the EEC parameters at different current density are presented in Supplementary Table 3. Here, R_C+A+BLs_ indicates combined resistance of the CEL, AEL and a combination of electrical double layers (EDLs) and diffusion boundary layers (DBLs). The C_SCJ_ and R_SCJ_ indicate charge storage (junction capacitance) and charge transfer resistance, which are direct indicators for the presence of water within the junction and water dissociation reaction resistance, respectively^6^.

### Electrochemical water dissociation performance and stability

Onset and steady water dissociation states, which are assessable by the analysis of *I*–*V* curves under reverse bias potential are two key indicators for the performance evaluation of a CBM^51^. The water dissociation test under reverse bias was performed within an electrochemical cell (Supplementary Fig. 9) composed of an anode, a cathode and two intermediate acid–base compartments beside a CBM of 3.2 cm^2^ active area (the AEL was kept facing the anode compartment). Both the intermediate compartments were filled with 0.5 M NaCl as a test solution. To avoid interference of the electrode reactions, two commercial cation exchange membranes (Nafion® 115) were placed between the intermediate and electrode compartments. Both the electrode compartments were filled with 0.5 M Na_2_SO_4_ solution as electrode rinse. Before measurements, the membranes were equilibrated in 0.5 M NaCl test solution for 24 h to minimise noises. Constant galvanostatic current steps were supplied with a DC power supply (HSPY-120-03, Trident Electronics Co., Ltd., China) through the platinum sheet electrodes (Tjaida, Tianjin China). Stable transmembrane voltage drops (after almost one minute at each DC-step) were recorded using a digital multimeter (VC890C^+^, VICTOR® YITENSEN^TM^), connected with two Ag/AgCl electrodes located at one millimetre apart from both surfaces of the CBM via Haber-Luggin capillaries (Supplementary Fig. 9). For water dissociation tests, the direct current density was increased from 0 to 109 mA cm^−2^, whereas the membrane durability and electrochemical performance stability were tested for five consecutive water dissociation cycles under loading, unloading and reloading of current densities up to 637 mA cm^−2^ during 4th and 5th cycles. Besides, the long-time electro-stability of the membranes was tested for 140 h (almost 6 days) at AUTOLAB workstation (PGSTAT 302N, Metrohm, Netherland) using the same electrochemical glass cell as for *I*–*V* test. The long-time electro-stability was performed in galvanostatic linear sweep voltammetry (LSV) mode, controlled by a computer with Nova 2.1.2 software. The LSV test is performed in continuously circulated 1 M NaCl as a test solution and 1 M NaSO_4_ solution as electrode rinse at 100 mA cm^−2^. During water dissociation and the cyclic and long-time membrane durability testing, solutions in all the four compartments were continuously circulated by peristaltic pumps at a flow rate of 30 mL min^−1^ to avoid concentration polarisation effects.

### Single-cell in-situ acid–base generation performance

Configuration of the CBM-integrated electrochemical single-cell for simultaneous water dissociation, desalination or treatment of concentrated inorganic salt solutions, and in-situ acid–base generation is shown in Fig. 6b. The AEL of the CBM having an effective surface area of 7.07 cm^2^ is orientated facing the anode and kept at the centre of the single-cell^52^. The CBM is surrounded by two sets of commercial Neosepta anion–cation exchange membranes (abbreviated as A and C in Fig. 6b). The single-cell is composed of the four types of compartments: 1-base, 2-acid, 3-auxiliary and 4-electrode, sequentially from the centre towards the cell electrodes. Four streams containing 200 mL of 0.5 M Na_2_SO_4_ salt solution were separately supplied and continuously circulated within each type of compartment. All the membranes and cell plates were separated by O-rings of silicone rubber to avoid electrolyte leakage. All the streams were continuously circulated using adjustable dual-headed peristaltic pumps with a constant flow rate of 30 mL min^−1^. All the membranes were equilibrated in the test solution for more than 24 h before the evaluation of the electrochemical performance. Water dissociation in the single-cell was investigated at 100 mA cm^−2^ direct current density (a typical value for industrial CBM-integrated electrochemical processes) using a power supply (FT6300A, Shenzhen Faith Technology Co., Ltd., China) through two electrodes made of titanium coated with ruthenium oxide. The applied direct current (DC potential) dissociates water into proton and hydroxide ion pairs within the catalytic junction (reaction zone), as exhibited in Fig. 1b.

The H^+^ ions permeate out through the CEL of the CBM, react with the sulphate ions (SO_4_^2−^) in the salt solution stream and generate H_2_SO_4_ in the acid compartment. Similarly, the OH^−^ ions permeate out through the AEL of the CBM and react with the available sodium ions (Na^+^) to produce NaOH in the base compartment^37^. The electrochemical water dissociation process was continued for 150 min and electrolyte samples from acid and base compartments were collected after specific time intervals (15, 30, 60, 90, 120 and 150 min) for further characterisations. The samples were very carefully titrated three times against standardised 0.009366 M NaOH and 0.010152 M HCl solutions using a mixed indicator (Bromocresol green plus methyl orange) and taken average of the three repeats. The desalination or depletion of salt Na^+^ ions $\left( {{\boldsymbol{M}}_{\boldsymbol{t}}^{{{{\mathbf{cations}}}}}} \right)$ from the acid compartment and SO_4_^2−^ ions $\left( {{\boldsymbol{M}}_{\boldsymbol{t}}^{{{{\mathbf{anions}}}}}} \right)$ from the base compartment was calculated by subtracting the consumed cations and anions (for the generation of base and acid) from their initial concentrations using Eqs. 2 and 3, respectively.

2 $$M_t^{{\mathrm{cations}}} = C_0^{M^{{\mathrm{cations}}}} - C_{tx}^{M^{{\mathrm{base}}}}$$

3 $$M_t^{{\mathrm{anions}}} = C_0^{M^{{\mathrm{anions}}}} - C_{tx}^{M^{{\mathrm{acid}}}}$$

The rates of acid and base generation are calculated using the following Eqs. 4 and 5, whereas the percentage depletion in salt cations and anions (% desalination) is calculated using the Eqs. 6 and 7, respectively.

4 $${\mathrm{The}}\,{\mathrm{arithmetic}}\,{\mathrm{average}}\,{\mathrm{generation}}\,{\mathrm{rate}}\,{\mathrm{of}}\,{\mathrm{the}}\,{\mathrm{acid}}\; \left( {{\mathrm{M}}\,{\mathrm{m}}^{ - {\mathrm{2}}}{\mathrm{min}}^{ - {\mathrm{1}}}} \right) = \mathop {\sum }\limits_{{\boldsymbol{x}} = 1}^6 \left\{ {\frac{{\left( {{\boldsymbol{C}}_{{\boldsymbol{tx}}}^{{\boldsymbol{M}}^{{{{\mathbf{acid}}}}}} - {\boldsymbol{C}}_0^{{\boldsymbol{M}}^{{{{\mathbf{acid}}}}}}} \right)}}{{{\boldsymbol{A}}_{\boldsymbol{m}} \times {\boldsymbol{t}}}}} \right\} \div 6$$

5 $${\mathrm{The}}\,{\mathrm{arithmetic}}\,{\mathrm{average}}\,{\mathrm{generation}}\,{\mathrm{rate}}\,{\mathrm{of}}\,{\mathrm{the}}\,{\mathrm{base}}\; \left( {{\mathrm{M}}\,{\mathrm{m}}^{ - 2}{\mathrm{min}}^{ - 1}} \right) = \mathop {\sum }\limits_{{\boldsymbol{x}} = 1}^6 \left\{ {\frac{{\left( {{\boldsymbol{C}}_{{\boldsymbol{tx}}}^{{\boldsymbol{M}}^{{{{\mathbf{base}}}}}} - {\boldsymbol{C}}_0^{{\boldsymbol{M}}^{{{{\mathbf{base}}}}}}} \right)}}{{{\boldsymbol{A}}_{\boldsymbol{m}} \times {\boldsymbol{t}}}}} \right\} \div 6$$

6 $${\mathrm{Percentage}}\,{\mathrm{depletion}}\,{\mathrm{of}}\,{\mathrm{the}}\,{\mathrm{salt}}\,{\mathrm{cation}}\; \left( {{\mathrm{\% }}\,{\mathrm{desalination}}} \right) = \frac{{\left( {{\boldsymbol{C}}_0^{{\boldsymbol{M}}^{{{{\mathbf{cation}}}}}} - {\boldsymbol{C}}_{{\boldsymbol{tx}}}^{{\boldsymbol{M}}^{{{{\mathbf{cation}}}}}}} \right)}}{{{\boldsymbol{C}}_0^{{\boldsymbol{M}}^{{{{\mathbf{cation}}}}}}}} \times 100$$

7 $${\mathrm{Percentage}}\,{\mathrm{depletion}}\,{\mathrm{of}}\,{\mathrm{the}}\,{\mathrm{salt}}\,{\mathrm{anion}}\; \left( {{\mathrm{\% }}\,{\mathrm{desalination}}} \right) = \frac{{\left( {{\boldsymbol{C}}_0^{{\boldsymbol{M}}^{{{{\mathbf{anion}}}}}} - {\boldsymbol{C}}_{{\boldsymbol{tx}}}^{{\boldsymbol{M}}^{{{{\mathbf{anion}}}}}}} \right)}}{{{\boldsymbol{C}}_0^{{\boldsymbol{M}}^{{{{\mathbf{anion}}}}}}}} \times 100$$

where *C*_0_ is the initial concentration and *C*_*tx*_ is the concentration of ions at the specific time (*t* = 15, 30, 60, 90, 120 and 150 min) of corresponding intervals (*x* = 1, 2, 3, 4, 5 and 6, respectively). The *A*_*m*_ indicates the area of CBM-ED cell in m^2^. The acid–base generation and salt-ions depletion (% desalination) in a single-cell (Fig. 6b) for Neosepta BP1 and SCBMs are presented in Fig. 6c, d.

Besides, the current efficiency (η) of a single-cell electrodialysis process was also evaluated by the generation of acid using the following Eq. 8.

8 $${\it{Percentage}}\,{\it{current}}\,{\it{efficiency}}\,({\it{\upeta }}) = \frac{{{\it{z}}({\it{C}}_{\it{t}} - {\it{C}}_0){\it{V}}_{\it{t}}{\it{F}}}}{{{\it{NIt}}}} \times 100{\mathrm{\% }}$$

where *C*_0_ and *C*_*t*_ are the molar concentrations of acid in the acid compartment at the beginning (*t* = 0 s) and the end of ED test (*t* = 150 min), respectively. *z* = 1 is the valency of the proton, *V*_*t*_ = 0.170 mL is the total volume of solution in the acid compartment at the end of ED test, *F* = 96,500 C mol^−1^ is the Faraday’s constant, *N* = 1 is the number of repeat units of the ED stack, *I* = 0.707 A is the applied current, and *t* = 9000 s is the time of ED test. The current efficiencies (η) of a single-cell electrodialysis process containing SCBMs, Neosepta BP1, and FumaTech FBM are presented in Supplementary Fig. 17.

## Supplementary information

Supplementary Information

Description of Additional Supplementary Files

Supplementary Data 1

Supplementary Data 2

## Data Availability

The data supporting the findings of this study are available within the paper, its Supplementary Information, and Supplementary Data 1 and 2. Other datasets analysed for the present study are available from the corresponding author on reasonable request.
