# Supplementary material for: Shielded goethite catalyst that enables fast water dissociation in bipolar membranes
Source: Nat Commun. 2021 Jan 4;12:9. doi: 10.1038/s41467-020-20131-1 (PMC7782813; doi:10.1038/s41467-020-20131-1)
Supplement: Supplementary file 1 — Supplementary Information [file 41467_2020_20131_MOESM1_ESM.pdf]

Supplementary Information for

Shielded goethite catalyst that enables fast water dissociation in bipolar membranes

Muhammad A. Shehzad[1,2,*], Aqsa Yasmin[1,2,*], Xiaolin Ge[1], Zijuan Ge[1], Kaiyu Zhang[1], Xian Liang[1], Jianjun Zhang[1], Geng Li[1], Xinle Xiao[1], Bin Jiang[3], Liang Wu[1], & Tongwen Xu[1]

[1]CAS Key Laboratory of Soft Matter Chemistry, Collaborative Innovation Centre of Chemistry for Energy Materials, Department of Applied Chemistry, School of Chemistry and Materials Science, University of Science and Technology of China, Hefei 230026, China

[2]Advanced Materials and Membrane Technology Center, Department of Polymer and Process Engineering, University of Engineering and Technology Lahore, G.T. Road-54890, Punjab Pakistan

[3]Department of Chemical Physics, School of Chemistry and Materials Science, University of Science and Technology of China, Hefei 230026, China

[*]These authors contributed equally.

Correspondence and requests for materials should be addressed to L.W. (email: liangwu8@ustc.edu.cn) or to T.W.X. (email: twxu@ustc.edu.cn)

Supplementary information contains **Pages**

- Supplementary Figures 1 to 18 2-16
- Supplementary Tables 1 to 7 17-43
- Supplementary Note 1 44
- Supplementary Discussion 44-47
- Supplementary References 48-49

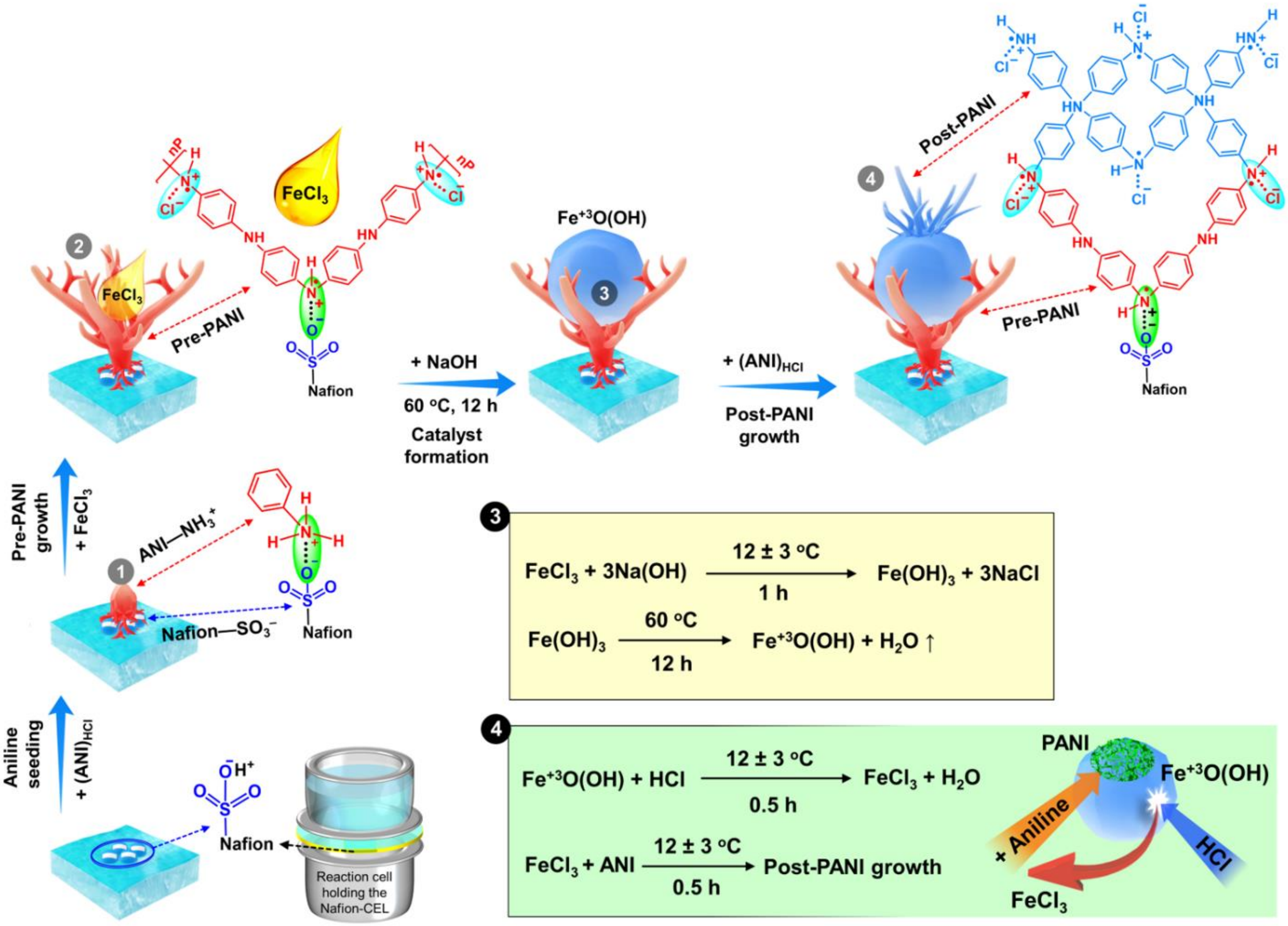

Supplementary Figure 1 | Schematic illustration for shielding catalyst and fabrication of the SCBMs. Stage 1, ANI seeding step, where aniline monomers embed as seeds at the Nafion-CEL surface by electrostatic interaction between aniline amine and Nafion sulfonate groups (Nafion—SO_3^-…^+H_3N—ANI). **Stage 2**, Polyaniline nanostructures nucleating from the aniline seeds, grow in such a way as to produce a porous networked web at the upper surface of Nafion-CEL, holding $FeCl_3$ solution. **Stage 3**, The $FeCl_3$ *in-situ* transform to $Fe(OH)_3$ within and at the pre-PANI porous web via the double displacement reaction ($FeCl_3 + 3NaOH \rightarrow Fe(OH)_3 + 3NaCl$). The $Fe(OH)_3$ is heat-treated at 60 ^oC for 12 h to produce goethite $Fe^{+3}O(OH)$ nanoparticles. **Stage 4**, An illustration indicating the shielding of catalyst nanoparticles. While the shielding process, HCl partially deteriorates the surface of $Fe^{+3}O(OH)$ nanoparticles and generates $FeCl_3$ which immediately polymerizes ANI at the nanoparticles and protects the $Fe^{+3}O(OH)$ particles beneath the post-PANI shield.

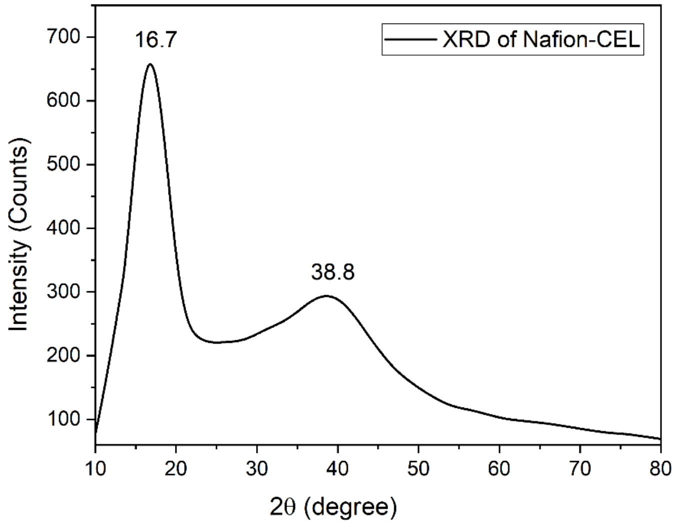

Supplementary Figure 2 │ XRD spectrum of bare Nafion-CEL exhibiting two wide bands at 2θ degrees of 16.7 and 38.8 as representative of the Nafion material.[1]

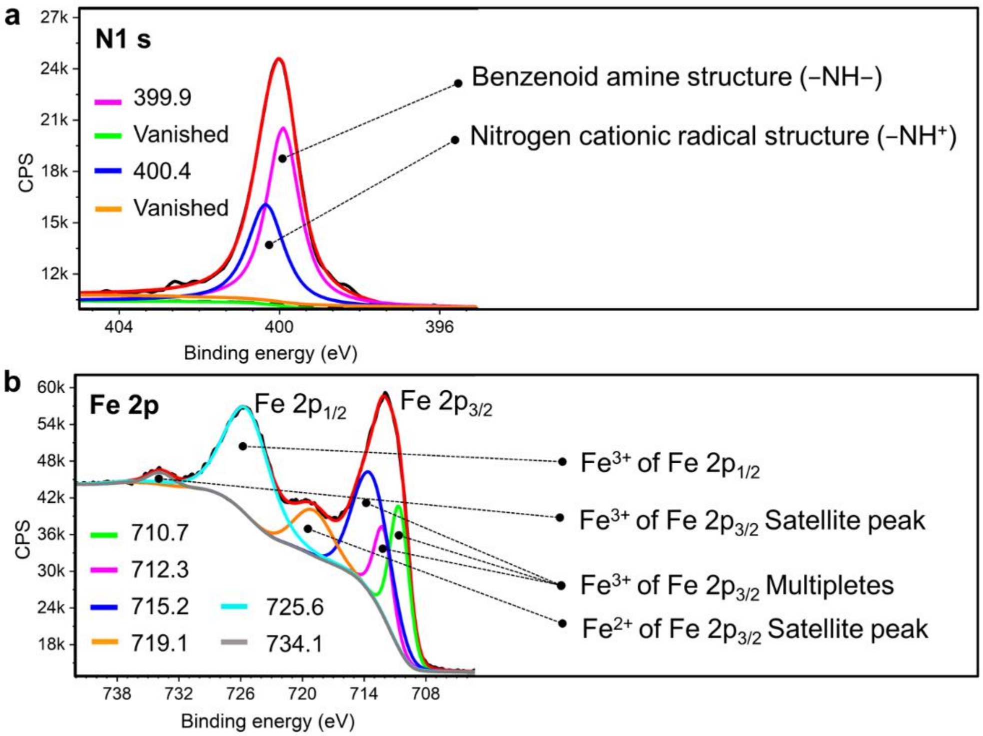

Supplementary Figure 3 │ The deconvoluted peaks in the core level XPS spectra of N 1s and Fe 2p. a, Loss of the two deconvoluted peaks (quinonoid imine, −NH= at 399.1 eV and nitrogen cationic radical, $-NH^{+}$ at 401.6 eV) in the core level XPS spectra of N 1s in the catalyst layer at pre-PANI shield indicating partial de-doping of the pre-PANI nanostructures after interaction with the added NaOH solution. Notably, the retrieval of these two peaks in the post-PANI shield (top Inset, Fig. 3a) is an indicator of the re-doping of polyaniline and recovery of electronically conductive characteristics of the PANI shields. **b**, The XPS core-level spectra of Fe 2p indicating the range of peaks (705 – 740 eV) and the deconvolved six representative peaks of the $Fe^{+3}O(OH)$ material.[2]

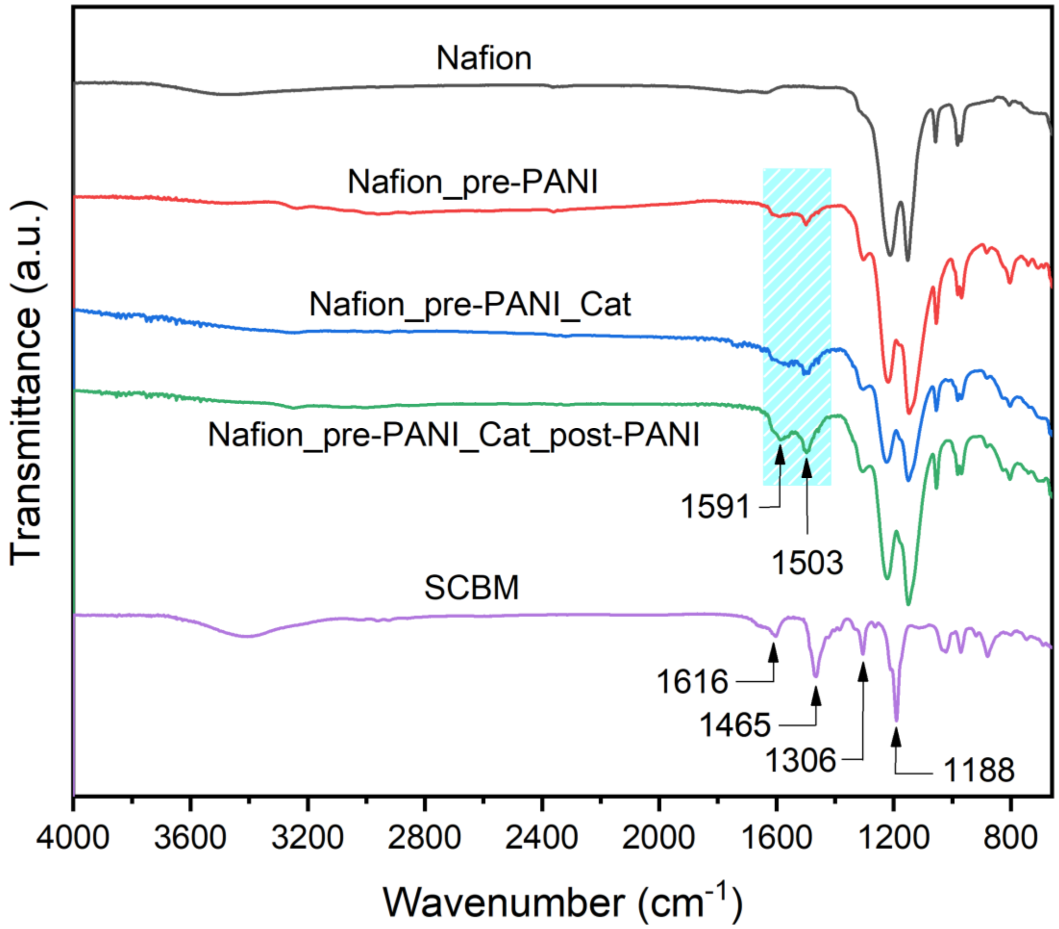

Supplementary Figure 4│ Chemical characterization of the membrane surfaces. FTIR-ATR spectra of the Nafion_pre-PANI (pre-PANI shield), Nafion_pre-PANI_Cat (catalytic layer), and Nafion_pre-PANI_Cat_post-PANI (shielded catalytic layer) compared with the bare Nafion-CEL are showing extra bands at 1591, and 1503 cm^{-1}. This change indicates the presence of PANI in its emeraldine salt state. Moreover, the disappearance of all the peaks related to the Nafion and shielded catalytic junction, whereas the appearance of new peaks of QPPO indicating successful fabrication of the shielded catalytic bipolar membranes (SCBMs).

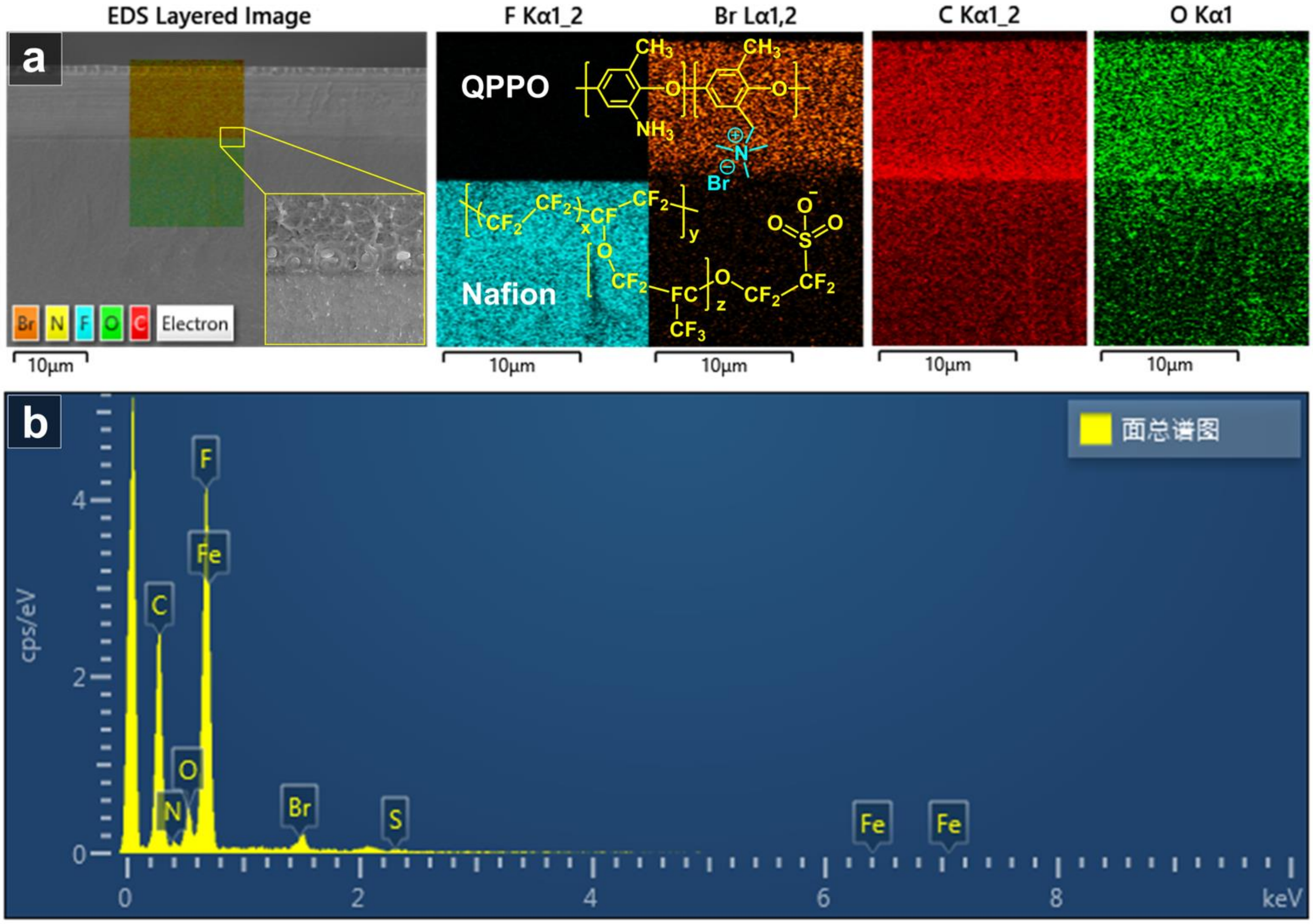

Supplementary Figure 5│ SEM-EDX analysis of the SCBMs indicates QPPO penetration into the junction. a, SEM cross-sectional image and the inset showing a unified SCBM and a clear shielded catalytic junction. Non-uniform and infused distribution of Br compared with F indicating the penetration of QPPO into the junction (up to Nafion-CEL surface). The junction containing excessive C of PANI and O of the catalyst indicating shielded catalyst at the junction. **b**, A graph of the energy dispersion showing the presence of a large amount of Fe (element of the catalyst) and N (element of the PANI), thus further confirming the shielded catalyst in the cross-section of the SCBMs.

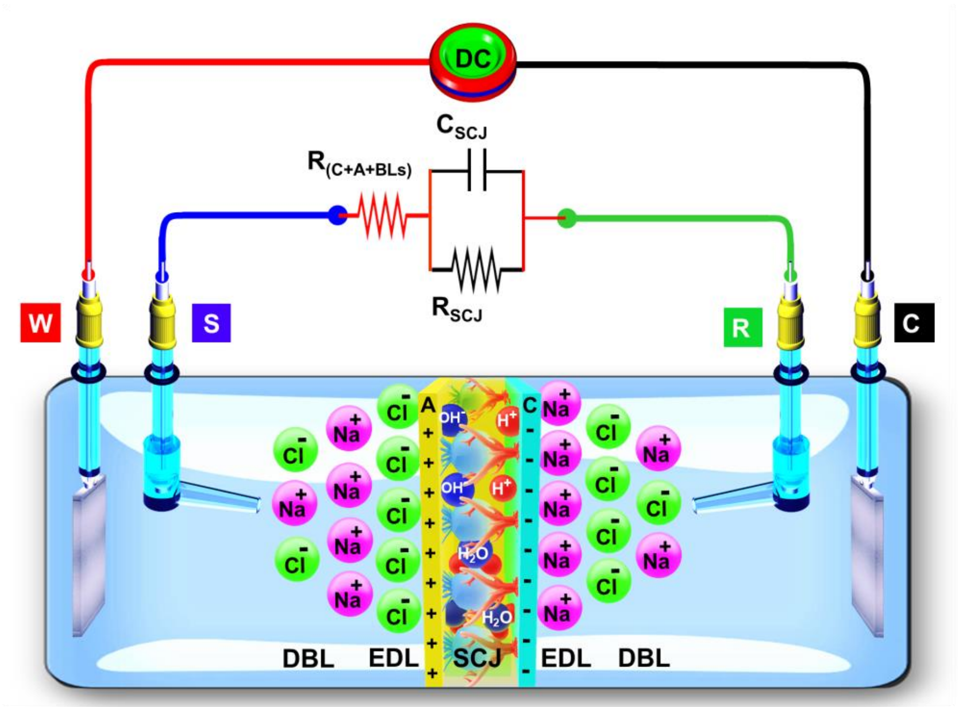

Supplementary Figure 6│ Configuration of an electrochemical cell for testing the EIS of the SCBMs. While the water dissociation and acid-base generation using SCBM-integrated electrochemical cell, solution ions neutralize the charges of membrane surfaces which produce electric double layers (EDLs) and diffusion boundary layers (DBLs) beside a membrane.[3] Herein, the electrochemical response of the surfaces and the junction of SCBM was evaluated under galvanostatic mode using Kelvin four-point sensing electrochemical cell connected with AUTOLAB workstation. The working (W) and counter (C) are platinum sheet electrodes, whereas the reference (R) and sense (S) are Ag/AgCl electrodes located about 1 mm on both sides of the bipolar membrane surface through Haber-Luggin capillaries. The recorded EIS data was simulated with an EEC model, $R_{C+A+BLs}(C_{SCJ}R_{SCJ})$ to interpret the quantitative response of the C/AEL and the SCJ. Here, the resistor $R_{(C+A+BLs)}$, C_{SCJ}, and R_{SCJ} are representing the cumulative CEL-AEL-BLs resistance, the junction capacitance, and water dissociation reaction resistance of the shielded catalytic junction (SCJ), respectively. Notably, the EIS testing was performed at AUTOLAB workstation under high stability mode and each experimental data point was taken as an average of 10 acquired points. The EIS tests were repeated three times at each current density and negligible variation in results was observed.

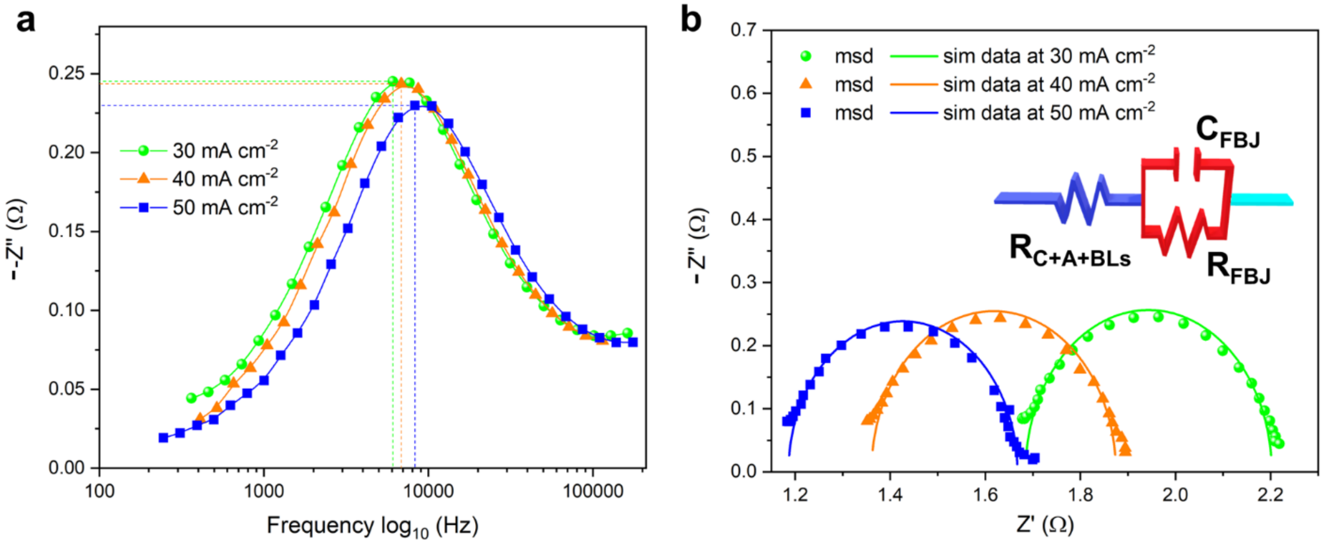

Supplementary Figure 7│ Electrochemical impedance spectroscopy (EIS) analysis of the FumaTech FBM. a, The EIS Bode curves indicating their slight shift towards lower impedance values, which preliminary predict a decrease in water-dissociation reaction resistance. **b**, The EIS Nyquist plots, where the measured EIS data (symbols) was simulated with the same EEC model (solid lines) as for SCBMs to interpret the response of the membrane and the junction. The Randles circuit model, $R_{C+A+BLs}(C_{FBJ}R_{FBJ})$, shows the fitting accuracy ~ 97% and Chi sq. value up to 10^{-4}). Here, the resistor $R_{C+A+BLs}$, the capacitor C_{FBJ} and the resistor R_{FBJ} representing the cumulative resistance of CEL-AEL-BLs, the junction capacitance, and junction resistance of the FumaTech FBM, respectively.[4]

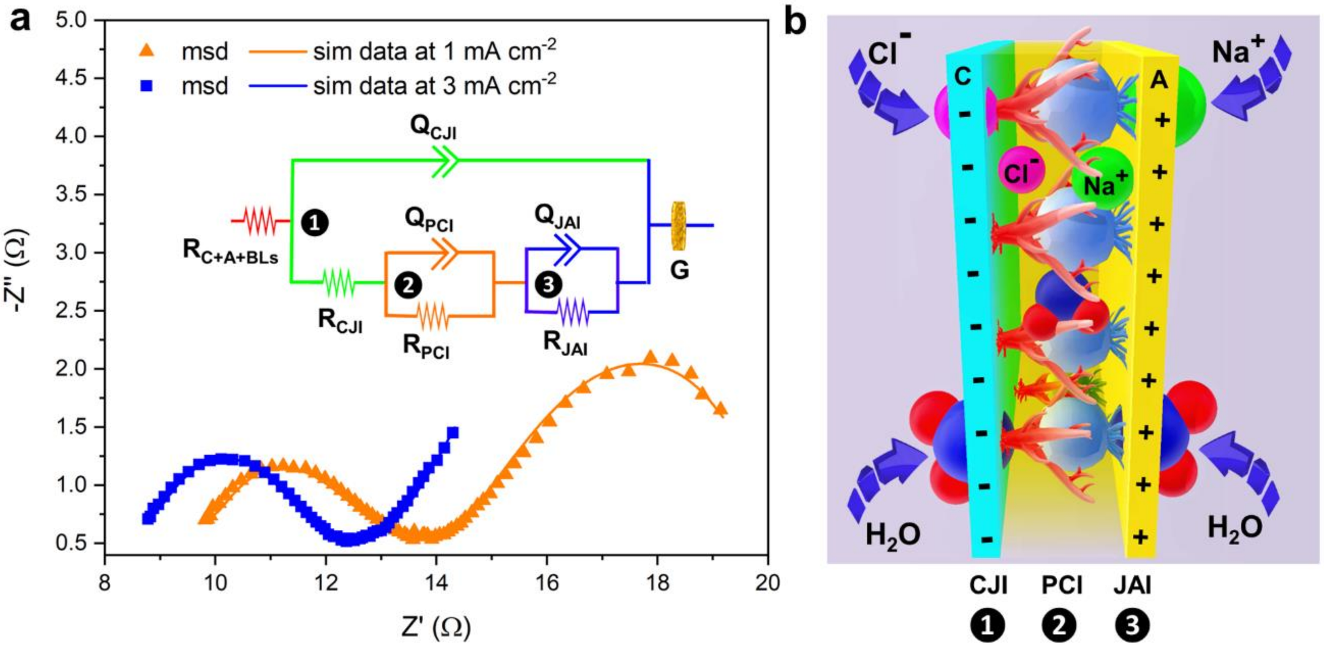

Supplementary Figure 8│ Galvanostatic EIS analysis of the SCBMs at low current densities. **a**, The EIS Nyquist plots, where the measured EIS data (symbols) was simulated with an EEC model (solid lines) to interpret the response of the C/AEL, and the components of SCJ. Inset showing a best-suited circuit model (fitting accuracy ~ 97% and Chi sq. value up to 10^{-4}), $R_{C+A+BLs}(Q_{CJI}(R_{CJI}(Q_{PCI}R_{PCI})(Q_{JAI}(R_{JAI}))))G$. Here, the resistor $R_{C+A+BLs}$ representing the cumulative CEL-AEL-BLs resistance and the G is the Gerischer element describing the water dissociation reaction within the bipolar junction. Q and R representing the constant phase element and the parallel resistor at the CEL-junction-interface (CJI) containing the pre-PANI shield (Q_{CJI} and R_{CJI}), PANI-catalyst-interface (PCI) containing the *in-situ* produced shielded goethite $Fe^{+3}O(OH)$ catalyst (Q_{PCI} and R_{PCI}), and junction-AEL-interface (JAI) comprising the post-PANI shield (Q_{JAI} and R_{JAI}). **b**, The illustrative representation of the SCBM at low current densities indicating hydration and co-ion permeation in the membrane.

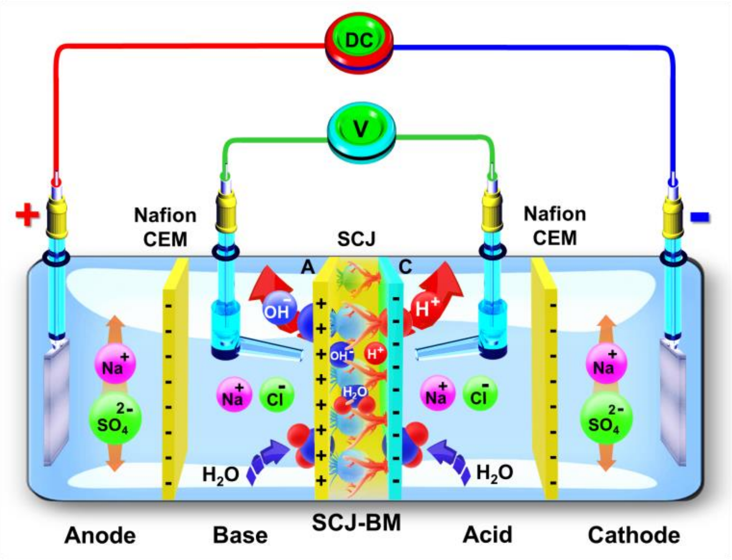

Supplementary Figure 9│ Configuration of an electrochemical cell for recording the galvanostatic current-voltage (I-V) curves and long-time electro-stability analysis. The cell was composed of four compartments, two electrode (anode and cathode), and two intermediate compartments. Here, direct current (DC stimuli) was supplied through platinum sheet anode and cathode, whereas trans-membrane voltage drop was recorded by a pair of Ag/AgCl electrodes located almost one millimetre away from the membrane surfaces through Haber-Luggin capillaries.

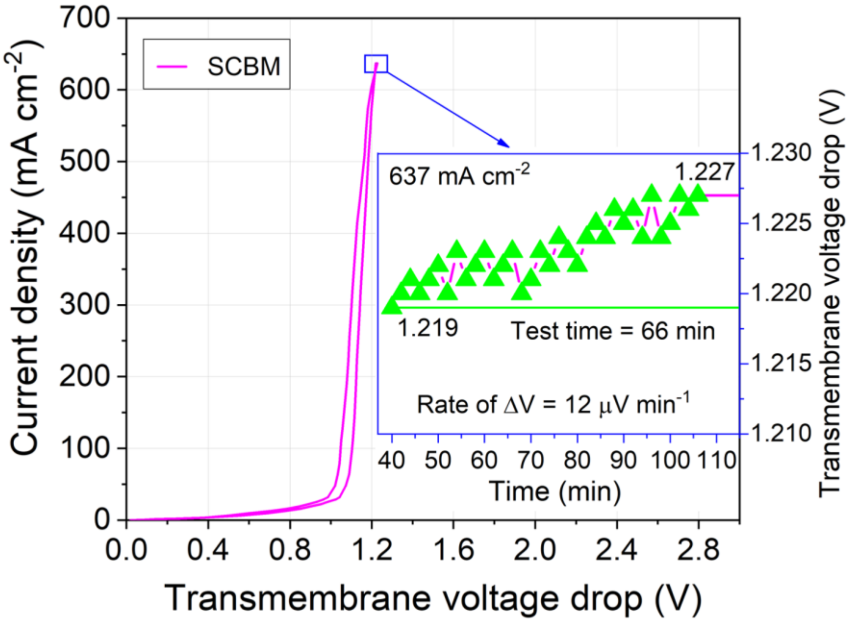

Supplementary Figure 10│ Electro-stability of the SCBMs at high operating current density. The electrochemical stability test of the SCBMs during loading up to 637 mA cm^{-2}, holding this high current density for 66 min, and then unloading current density to zero. A very slow rate of change in voltage increase (12 μV min^{-1}) and highly reversible trend in the I-V cycle is indicative of good electrochemical stability of SCBMs during fast water dissociation at high operating current densities.

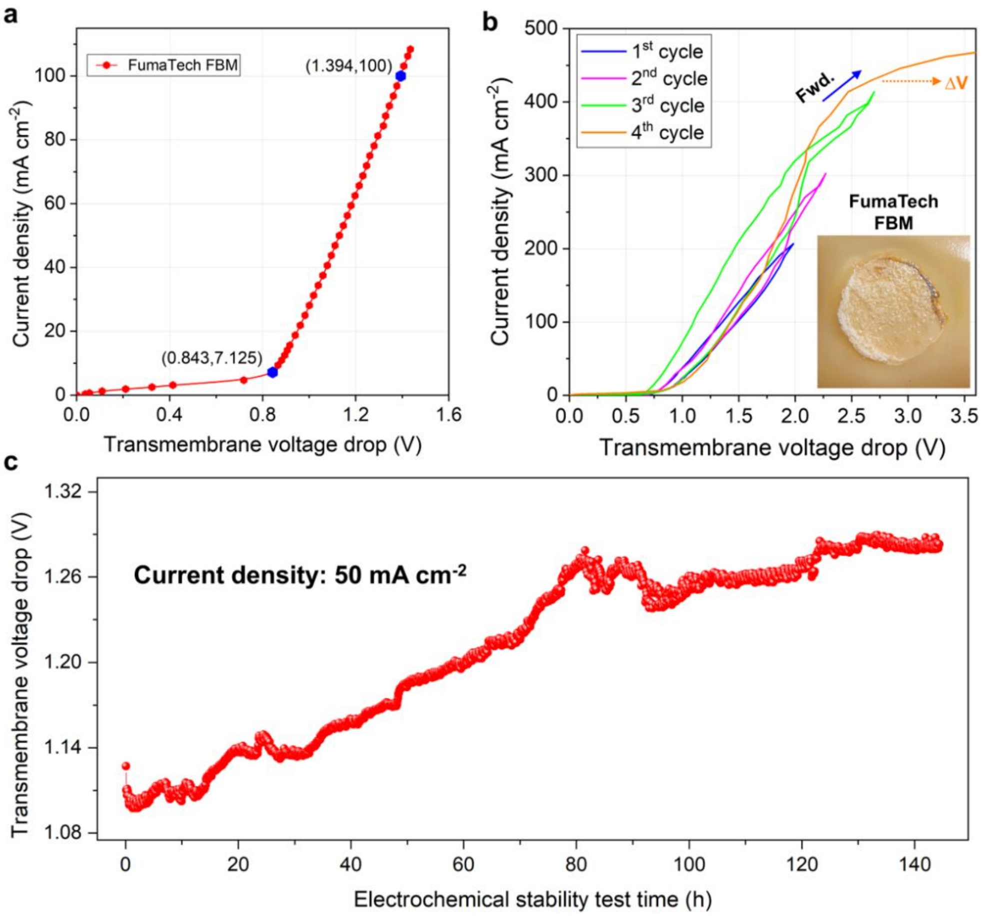

Supplementary Figure 11 | Galvanostatic polarization analysis (I-V curves) and stability of FumaTech FBM. a, The polarization I-V curve up to 109 mA cm^{-2} although indicating the requirement of only 0.843 V transmembrane potential drop at limiting current density (U_{LCD}). But, larger transmembrane potential drop at 100 mA cm^{-2} current density (U_{100}: 1.394 V) than the commercial Neosepta BP1 (U_{100}:1.3 V) and the fabricated SCBMs (U_{100}: 1.1 V) indicates the superiority of the shielding and in-situ catalyst formation strategy. **b**, The four I-V cycles (1st - 4th) were recorded at loading-unloading-reloading current densities between 0 to 207, 303, 414, and 460 mA cm^{-2}. Initial three cycles (1st – 3rd) although showing direct relation of the voltage with the loading-unloading-reloading of the current densities but the excessive voltage lag is indicating poorer stability of the FumaTech FBM at increased water dissociation current densities. Moreover, failure of the cyclic reversibility at 460 mA cm^{-2} (4th cycle) and severe membrane damage (Inset showing a digital photograph) is indicating inferior electro-stability of the FBM than the commercial Neosepta BP1 (sufficiently stable) and the fabricated SCBM (excellently stable and reversible with negligible voltage lag). **c**, Long-time electro-stability (6 days, 145 h) of the FBM membrane during water dissociation at 50 mA cm^{-2}. Notably, we were unable to test the long-time electro-stability at 100 mA cm^{-2} due to a large transmembrane voltage drop within the test time which is beyond the voltage limitations of the AUTOLAB electrochemical workstation (PGSTAT 302N, Metrohm, Netherland). The stability data indicating a large increase in transmembrane voltage drop with prolonged testing time and a quicker decline in water dissociation rate of the FBM compared with Neosepta BP1 and the SCBMs.

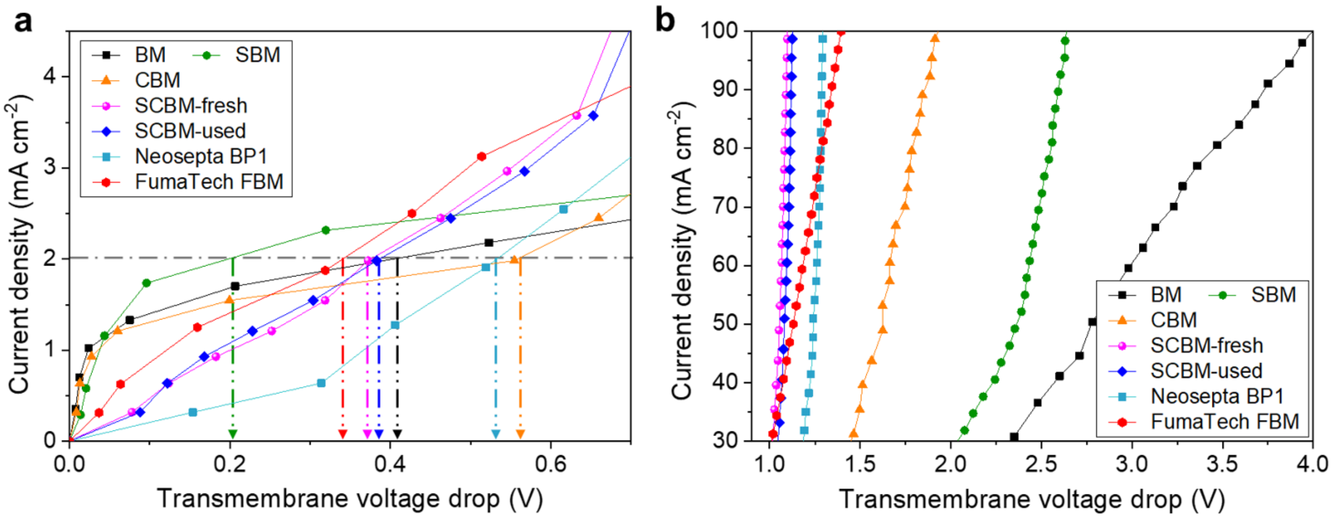

Supplementary Figure 12│ The I-V curves in a low and high current-density range. a, The water dissociation measurements are usually affected at low current density due to co-ion leakage across the bipolar membranes which leads to a parasitic or charge-compensating current. The I-V curves at a low current density (2 mA cm^{-2}) indicating varied transmembrane voltage drop for all the fabricated and commercial bipolar membranes in this work. Herein, the voltage drop in the representative SCBM-fresh and SCBM-used membranes is almost comparable with the FumaTech FBM and much lower than the Neosepta BP1 at the same current density. **b**, At high current densities this leakage current becomes negligible in the SCBMs compared with all other bipolar membranes, as observable from the negligible voltage drop at increased current density.

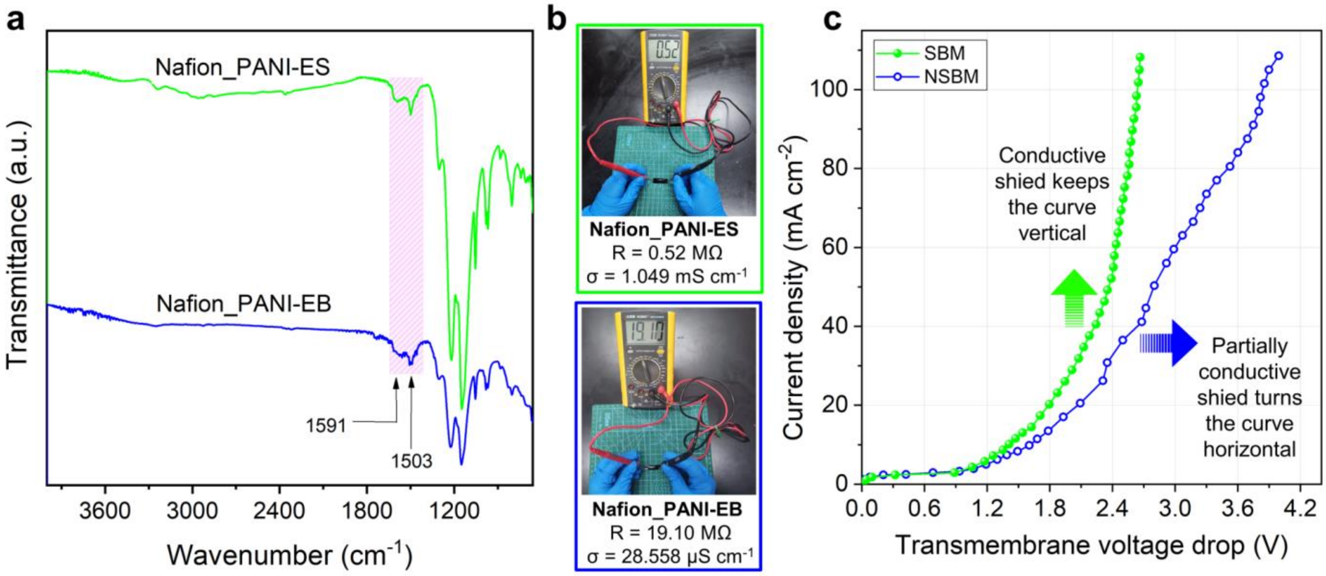

Supplementary Figure 13│ Electrochemical analysis indicates improved water dissociation with a conductive polyaniline shield. **a**, FTIR-ATR spectra of the *in-situ* grown polyaniline on Nafion-CEL in its two states such as conductive emeraldine salt (Nafion_PANI-ES) and non-/less conductive emeraldine base (Nafion_PANI-EB). **b**, Two-probe resistance testing meter confirms high conductivity of Nafion_PANI-ES and comparatively very low conductivity of Nafion_PANI-EB. **c**, Electrochemical I-V curves of the fabricated bipolar membranes comprising conductive polyaniline shield (SBMs) and non-conductive polyaniline shield (NSBMs) approving beneficial role of the conductive polyaniline such as the near-vertical trend in I-V curve.

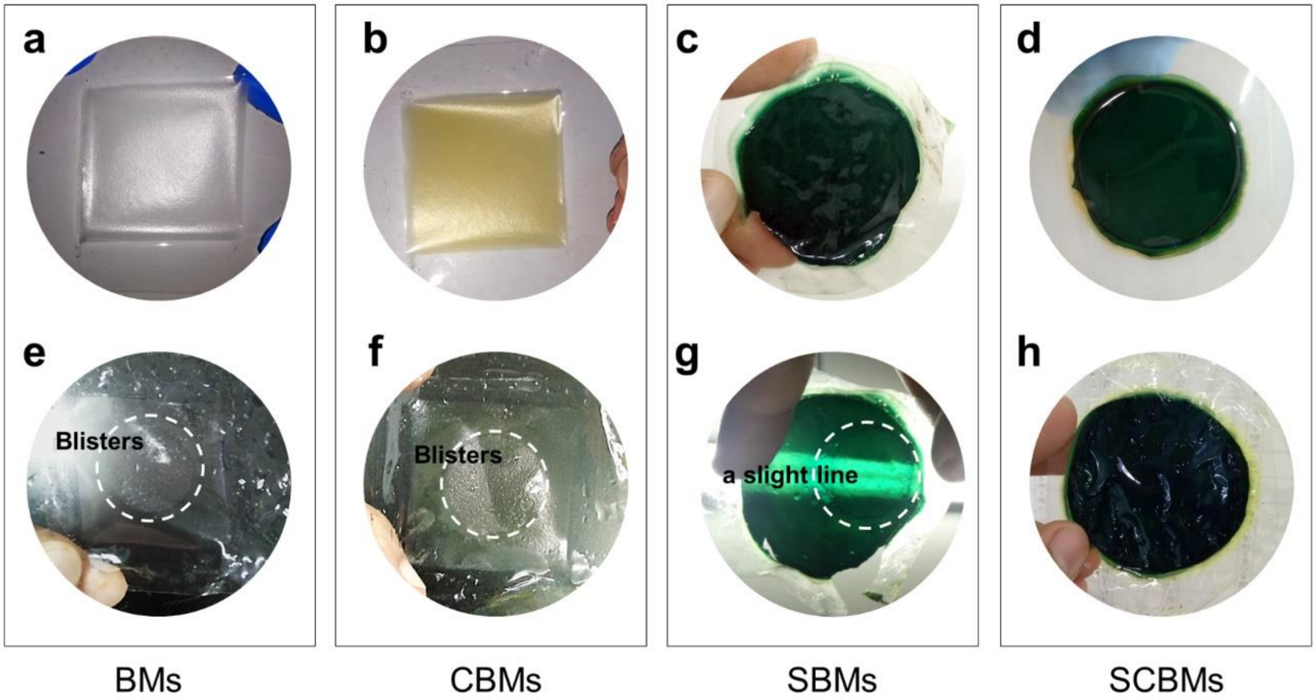

Supplementary Figure 14 │ Digital photographs of the bipolar membranes. a-d, and **e-h** are the bipolar membranes before and after the galvanostatic I-V testing up to 109 mA cm^{-2} testing current density for the bipolar membranes without catalyst (BMs), containing only the *ex-situ* synthesized catalyst (CBMs), containing only the PANI shields (SBMs), and containing the *in-situ* synthesized shielded catalyst (SCBMs). The BMs and CBMs which are fabricated by the conventional spray deposition of the catalyst with Siansonic ultrasonic spray deposition machine and solution casting of the C/AEL could not remain stable, the appearance of blisters during the I-V testing. Whereas, the shielded BMs and the shielded catalytic BMs (SBMs and SCBMs) which are fabricated via the proposed *in-situ* junction fabrication pathways remained highly stable during the I-V testing. Notably, the horizontal white patch in photograph **g** is a tube-light behind the membrane to enlighten the slight damage (circled line inside the marked white circle) in the SBM during the polarization test.

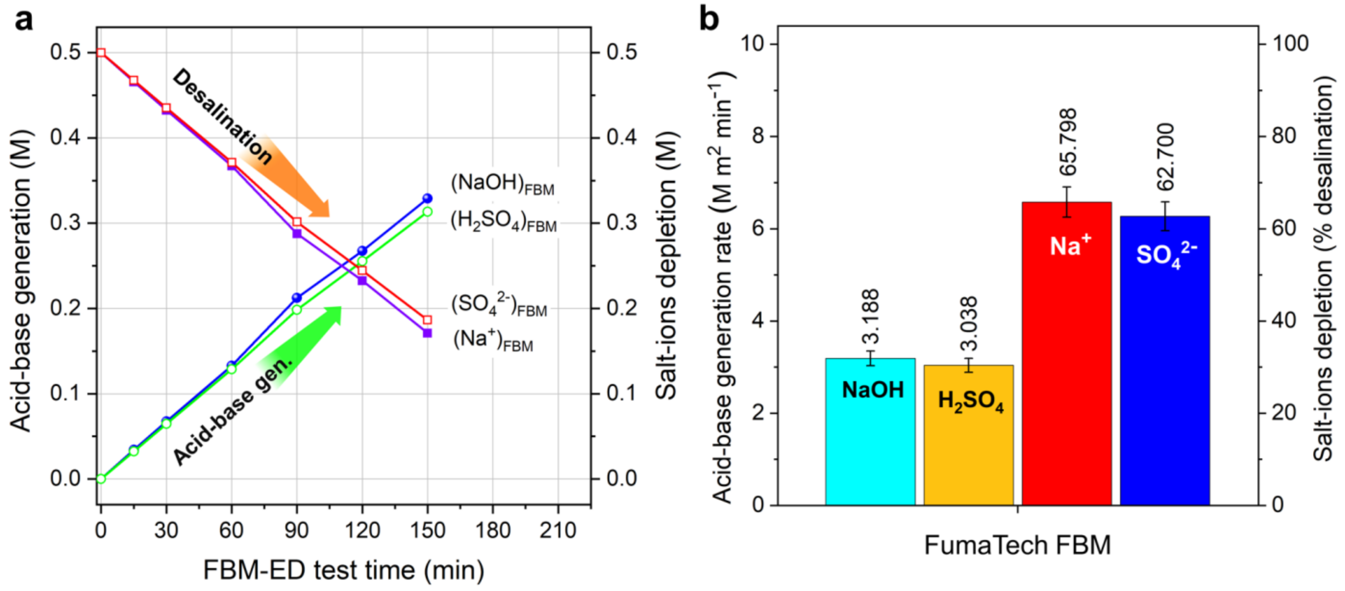

Supplementary Figure 15│ Electrodialysis performance of FumaTech FBM (FBM-ED). **a**, Acid-base generation and saline-water desalination performance with the increased FBM-ED test time. **b**, The acid-base generation rate and the percentage saline water desalination (of commercial FumaTech FBM (66% of Na^+ and 63% of SO_4^{2-} salt-ions depletion along with only 3.038 M m^{-2} min^{-1} of H_2SO_4 and 3.188 M m^{-2} min^{-1} of NaOH which are within data variation of ± 5 % s.d.) is slightly better than the performance of commercial Neosepta BP1 (only 61% of Na^+ and 54% of SO_4^{2-} salt-ions depletion along with only 2.577 M m^{-2} min^{-1} of H_2SO_4 and 3.105 M m^{-2} min^{-1} of NaOH generations, Fig. 6d). However, the ED performance of FBM and BP1 are significantly lower than the ED performance of SCBMs, enabling the depletion of 84% Na^+ and 77% SO_4^{2-} salt-ions within only 150 min and fast generation rate of H_2SO_4 (3.931 M m^{-2} min^{-1}) and 4.395 M m^{-2} min^{-1} of NaOH (Fig. 6d).

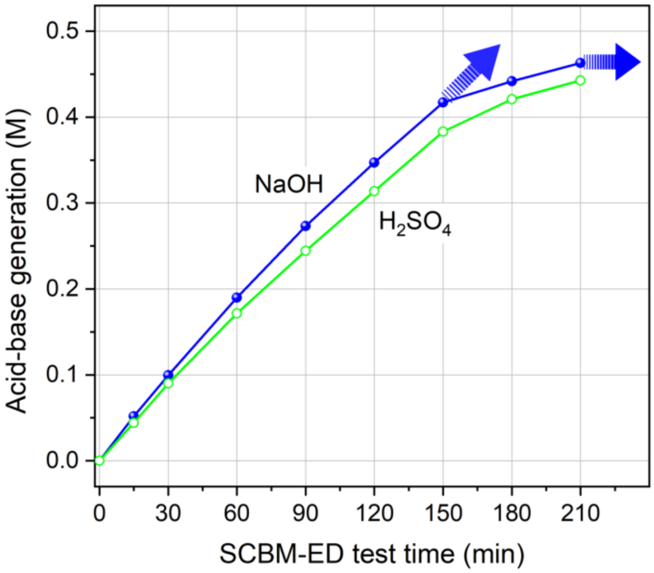

Supplementary Figure 16│ SCBMs-integrated ED process (SCBMs-ED) for an extended time of 210 min. The SCBMs-ED for an extended time exhibiting an abrupt decrease in the generation of acid and base after 150 min of the process due to a single-cell and lab-scale ED setup of the area only 7.07 cm^2, 0.5 M concentration of the mineral NaCl solutions, and consumption of excessive salt ions in the feed streams. Therefore, a test time of 150 min is chosen for all the ED tests.

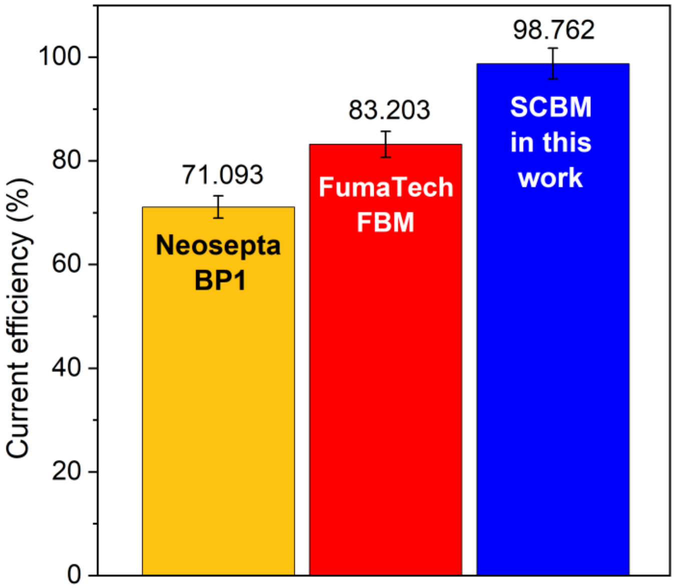

Supplementary Figure 17│ Current efficiency of bipolar membranes integrated single-cell electrodialysis (ED) process. The SCBMs-integrated ED process showing maximum current efficiency compared with the commercial FumaTech FBM and Neosepta BP1 membranes at the same operational conditions. The results are within data variation of ±3 % s.d.

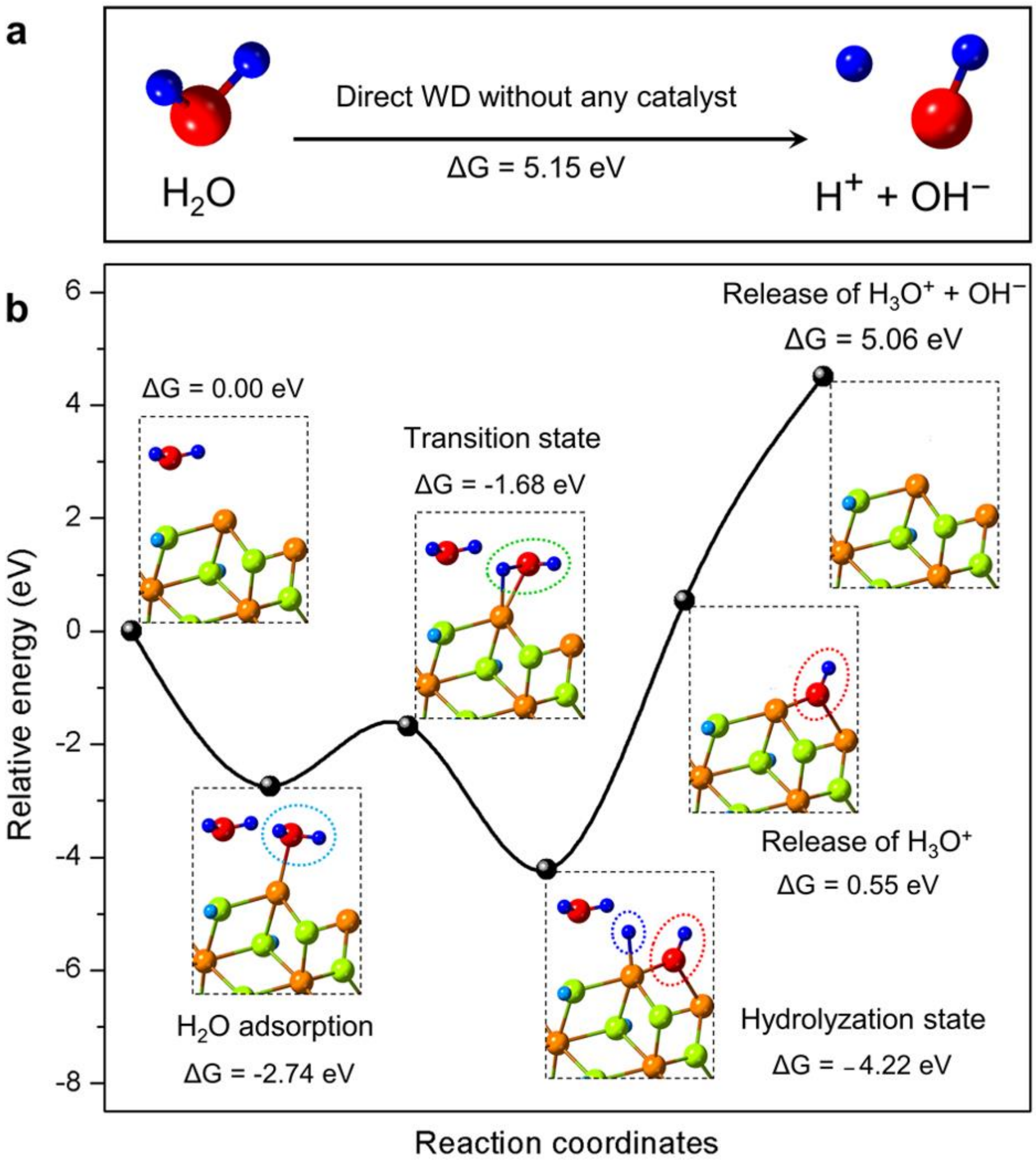

Supplementary Figure 18 | Relative Gibbs free energies of the water dissociation. a, Schematic illustrating relative Gibbs free energy required for direct water dissociation without the use of any catalyst. **b,** The graphical representation and the schematics showing DFT-calculated relative Gibbs free energies for various reaction coordinates at the goethite $Fe^{+3}O(OH)$ catalyst surface. An almost the same overall energy for water dissociation in both the cases ($\Delta G_{direct} \approx 5.15$ eV and $\Delta G_{indirect} \approx 5.06$ eV) is due to the energy conservation law. However, a sufficiently lower activation energy barrier at the transition state of the synthesized catalyst than several famous catalytic materials in Fig. 3c exhibiting the significance of the reported goethite $Fe^{+3}O(OH)$ catalyst. Notably, the release of the produced ions (H^+ (as H_3O^+) and OH^-) from the catalyst surface and the catalytic junction can be assisted by externally applied electrical energy (second Wein effect).[5-7]

Supplementary Table 1 | The percentage elemental composition of C, N, and S obtained by elemental combustion analysis for three samples of ANI-seeded Nafion-CEL. The average values are indicating that almost 18 wt % of S (Nafion—SO_3^-) has electrostatically interacted with N (^+H_3N—ANI) and the resulting salt act as ANI seeds for subsequent heterogeneous growth of PANI.

Samples	weight (mg)	C (%)	N (%)	S (%)
1	1.665	26.53	0.383	2.191
2	1.857	29.681	0.446	2.445
3	1.869	27.799	0.451	2.458
Average	1.797	28.003	0.427	2.365
Percentage of the Nitrogen entrenched on Sulphur		$\left(\frac{0.427}{2.365}\right) \times 100 \approx 18\ \%$		

Supplementary Table 2 | Zeta-potential measurements indicating electrostatic binding of the *in-situ* grown PANI with the Nafion-CEL and porous morphology of the PANI surface layer due to increased value of the zeta-potential and the asymmetry. The measurements for both the membranes show almost the same correlation factor (chi^2: 0.9645) or fitting accuracy.

	**Nafion-CEL**				**Nafion-PANI**			
Sr	pH	Zeta-Potential mV	Asymmetry mV	Correlation (chi^2)	pH	Zeta-Potential mV	Asymmetry mV	Correlation (chi^2)
1	4.932	-0.716	-5.748	0.935	4.927	-0.139	-1.994	0.953
2	4.931	-1.079	-4.349	0.961	4.934	-0.426	-1.564	0.969
3	4.929	-0.757	-4.624	0.972	4.929	-0.447	-0.906	0.956
4	4.931	-1.256	-2.974	0.998	4.929	-0.349	-0.950	0.972
The following row contains the average values of all the above parameters.								
	4.931	**-0.952**	-4.424	0.966	4.931	**-0.340**	-1.353	0.963

Supplementary Table 3│ Electrochemical properties of the C/AEL and the SCJ of SCBM including the junction thickness (λ_{SCJ}), junction capacitance (C_{SCJ}), and water dissociation reaction resistance within the junction (R_{SCJ}) at various current densities (I_d). The simulation of electrochemical impedance spectroscopy (EIS) data showed more than 97 % fitting accuracy.[3]

I_d (mA cm^{-2})	λ_{SCJ} (nm)	$R_{(C+A+BLs)}$ (Ω cm^2)	C_{SCJ} (μF cm^2)	R_{SCJ} (Ω cm^2)	St. error (%)	Chi sq., χ^2 values
30	83.4	0.912	18.7	0.679	2.459	6.047×10^{-04}
40	90.5	0.906	21.1	0.627	1.838	3.377×10^{-04}
50	93.1	0.899	21.9	0.549	2.033	4.134×10^{-04}

Supplementary Table 4│ Electrochemical properties of the C/AEL and the junction of FumaTech FBM. The data showing the junction thickness (λ_{FBJ}), junction capacitance (C_{FBJ}), and water dissociation reaction resistance within the junction (R_{FBJ}) at varied current densities (I_d). The simulation of electrochemical impedance spectroscopy (EIS) data showed more than 97 % fitting accuracy.[3]

I_d (mA cm^{-2})	λ_{FBJ} (nm)	$R_{(C+A+BLs)}$ (Ω cm^2)	C_{FBJ} (μF cm^2)	R_{FBJ} (Ω cm^2)	St. error (%)	Chi sq., χ^2 values
30	10.4	1.686	4.773	0.519	1.69	2.858×10^{-04}
40	11.7	1.362	4.906	0.510	1.615	2.607×10^{-04}
50	13.6	1.187	5.171	0.479	2.139	4.574×10^{-04}

Supplementary Table 5 | The fitted-simulation results for EEC modelling parameters of the impedance spectra, shown in Supplementary Fig. 8.

EEC Parameters	$R_{C+A+BLs}\left(Q_{CJI}\left(R_{CJI}(Q_{PCI}R_{PCI})\left(Q_{JAI}(R_{JAI})\right)\right)\right)G$	
	EIS of SCBMs at 1 mA cm^{-2} Chi sq., χ^2 values: 9.722E-4 St. error < 3.118%	EIS of SCBMs at 3 mA cm^{-2} Chi sq., χ^2 values: 4.895E-4 St. error < 2.212%
$R_{C+A+BLs}$ (Ω cm^2)	9.493	8.647
Q-Y_{CJI} (S · cm^{-2} s^n)	0.002	0.013
Q-n_{CJI}	0.726	0.913
R_{CJI} (Ω cm^2)	1.961	0.488
Q-Y_{PCI} (S · cm^{-2} s^n)	0.043	0.97
Q-n_{PCI}	0.603	0.798
R_{PCI} (Ω cm^2)	1.293	1.044
Q-Y_{JAI} (S · cm^{-2} s^n)	1.592E-4	1.071E-3
Q-n_{JAI}	0.873	0.719
R_{JAI} (Ω cm^2)	1.779	1.48
G-Yo (S · s$^{0.5}$)	5.563	6.914
G-Ka (s^{-1})	0.941	0.984

Supplementary Table 6 | Comparison of electrochemical characteristics shows sufficiently low limiting current density (LCD), smaller potential drop at the LCD (U_{LCD}) and steady water dissociation at 100 mA cm^{-2} (U_{100}) of the fabricated SCBMs in this work with the recently published 2-dimensional CBMs and commercial bipolar membranes.

Membranes	catalysts	Testing conditions	LCD (mA cm^{-2})	U_{LCD} (V)	U_{100} (V)	Ref.
**SCBM**	**PANI shielded $Fe^{+3}O(OH)$**	**0.5 M NaCl**	**6**	**0.8**	**1.1**	**This work**
FumaTech FBM	Commercial catalyst	0.5 M NaCl	7.1	0.843	1.394	This work[8]
Neosepta BP1	Commercial catalyst	0.5 M NaCl	9	1.12	1.297	This work[9]
BM-2	Silane groups	0.5 M Na_2SO_4	34.0	2.1	2.56	[9]
Boltorn H30	─COOH functionalities	0.5 M Na_2SO_4	18.0	1.9	3.85	[10]
BM-2	Silane groups	0.5 M Na_2SO_4	34.0	2.1	2.56	[9]
BPM-SiOH	SiOH	2.0 M NaCl	17	4.9	8.96	[11]
LbL-BPM	PEDOT:PSS/PEI/FAA	2 M NaCl	10	0.95	2.5	[12]
LbL-BPM	GO/PEDOT	1 M NaCl	3	1.5	2.2	[13]
GO-BPM	Graphite oxide	0.5 M HCl & 0.5 M KOH	3.0	0.87	1.45	[3]
BiOCl/BPM	BiOCl	0.3 M Na_2SO_4	9 - 10	2.6 - 3.0	5.9 - 5.2	[14]
MIL-BPM	FeMIL-101-NH_2	0.5 M NaCl	19-23	2.6 - 3.8	4.2 - 5.9	[15]
Crosslinked BPM	PEI/GA/PVA	0.5 M Na_2SO_4	11	0.9	1.79	[16]
BPM-LYS	Lysozyme	0.10 M NaCl	62.9	2.87	5.5	[17]

EBPM-2	Polyethylene glycol (PEG)	0.10 M NaCl	10.5	1.7	2.4	[18]
BPM-GO	Graphene oxide	0.10 M NaCl	9	3.8	5	[19]
BPM-BSA	Bovine serum	0.10 M NaCl	56.4	3.22	7.9	[17]
BPM-TiOH	TiOH	2.0 M NaCl	16	4.8	11.2	[11]
BPM-3D	$Al(OH)_3$	0.5 M Na_2SO_4	5.9	0.78	0.95	[8]
BPM-2D	$Al(OH)_3$	0.5 M Na_2SO_4	5.5	0.86	1.2	[8]
BPM-ZrOH	ZrOH	2.0 M NaCl	19	3.7	10.1	[11]
Composite BPM	RCOONa	0.5 M NaCl	3.75	2.02	-	[20]
GQDs–Cu_2O	Graphene quantum dots–Cu_2O	0.5 M Na_2SO_4	20	2.9	4.5	[21]
Irradiated CBM	PVA–CMC/CeO_2–CS	1 M Na_2SO_4	10	3.5	4.9	[22]

Supplementary Table 7 | Cell contents for the density functional theory (DFT) calculations.

Original state				
	Atom	Fractional coordinates of atoms		
Element	number	u	v	w
------------	------------	------------	------------	------------
H	1	0.304455	0.153890	0.019177
H	2	0.304455	0.069337	0.175775
H	3	0.304455	0.984784	0.332373
H	4	0.054455	0.811151	0.046079
H	5	0.054455	0.726598	0.202677
H	6	0.054455	0.642045	0.359274
H	7	0.195545	0.268874	0.124378
H	8	0.195545	0.184321	0.280976
H	9	0.195545	0.099768	0.437573
H	10	0.445545	0.611614	0.097476
H	11	0.445545	0.527061	0.254074
H	12	0.445545	0.442508	0.410672
H	13	0.804455	0.153890	0.019177
H	14	0.804455	0.069337	0.175775
H	15	0.804455	0.984784	0.332373
H	16	0.554455	0.811151	0.046079
H	17	0.554455	0.726598	0.202677
H	18	0.554455	0.642045	0.359274
H	19	0.695545	0.268874	0.124378
H	20	0.695545	0.184321	0.280976
H	21	0.695545	0.099768	0.437573
H	22	0.945545	0.611614	0.097476
H	23	0.945545	0.527061	0.254074
H	24	0.945545	0.442508	0.410672
H	25	0.316066	0.124099	0.550375
H	26	0.222880	0.235028	0.532640
H	27	0.818144	0.250629	0.547860

H	28	0.769569	0.379976	0.530451
H	29	0.328466	0.428234	0.563351
H	30	0.249636	0.564117	0.569707
H	31	0.350206	0.747286	0.593285
H	32	0.266130	0.872447	0.568379
O	33	0.132800	0.041782	0.000000
O	34	0.132800	0.957229	0.156598
O	35	0.132800	0.872676	0.313196
O	36	0.407756	0.181488	0.023898
O	37	0.407756	0.096935	0.180496
O	38	0.407756	0.012382	0.337094
O	39	0.382800	0.923259	0.065256
O	40	0.382800	0.838706	0.221854
O	41	0.382800	0.754153	0.378452
O	42	0.157756	0.783553	0.041358
O	43	0.157756	0.699000	0.197956
O	44	0.157756	0.614447	0.354554
O	45	0.367200	0.380983	0.143555
O	46	0.367200	0.296430	0.300153
O	47	0.367200	0.211877	0.456751
O	48	0.092244	0.241276	0.119657
O	49	0.092244	0.156723	0.276255
O	50	0.092244	0.072170	0.432852
O	51	0.117200	0.499506	0.078299
O	52	0.117200	0.414953	0.234897
O	53	0.117200	0.330400	0.391495
O	54	0.342244	0.639212	0.102197
O	55	0.342244	0.554659	0.258795
O	56	0.342244	0.470106	0.415393
O	57	0.632800	0.041782	0.000000
O	58	0.632800	0.957229	0.156598
O	59	0.632800	0.872676	0.313196

O	60	0.907756	0.181488	0.023898
O	61	0.907756	0.096935	0.180496
O	62	0.907756	0.012382	0.337094
O	63	0.882800	0.923259	0.065256
O	64	0.882800	0.838706	0.221854
O	65	0.882800	0.754153	0.378452
O	66	0.657756	0.783553	0.041358
O	67	0.657756	0.699000	0.197956
O	68	0.657756	0.614447	0.354554
O	69	0.867200	0.380983	0.143555
O	70	0.867200	0.296430	0.300153
O	71	0.867200	0.211877	0.456751
O	72	0.592244	0.241276	0.119657
O	73	0.592244	0.156723	0.276255
O	74	0.592244	0.072170	0.432852
O	75	0.617200	0.499506	0.078299
O	76	0.617200	0.414953	0.234897
O	77	0.617200	0.330400	0.391495
O	78	0.842244	0.639212	0.102197
O	79	0.842244	0.554659	0.258795
O	80	0.842244	0.470106	0.415393
O	81	0.231927	0.168830	0.571149
O	82	0.731615	0.305314	0.556394
O	83	0.236041	0.474267	0.557292
O	84	0.280561	0.780262	0.558469
Fe	85	0.482055	0.368145	0.055828
Fe	86	0.482055	0.283592	0.212426
Fe	87	0.482055	0.199039	0.369024
Fe	88	0.232055	0.596896	0.009428
Fe	89	0.232055	0.512343	0.166026
Fe	90	0.232055	0.427790	0.322624
Fe	91	0.017945	0.054619	0.087727

Fe	92	0.017945	0.970066	0.244325
Fe	93	0.017945	0.885514	0.400923
Fe	94	0.267945	0.825869	0.134127
Fe	95	0.267945	0.741316	0.290725
Fe	96	0.267945	0.656763	0.447323
Fe	97	0.982055	0.368145	0.055828
Fe	98	0.982055	0.283592	0.212426
Fe	99	0.982055	0.199039	0.369024
Fe	100	0.732055	0.596896	0.009428
Fe	101	0.732055	0.512343	0.166026
Fe	102	0.732055	0.427790	0.322624
Fe	103	0.517945	0.054619	0.087727
Fe	104	0.517945	0.970066	0.244325
Fe	105	0.517945	0.885514	0.400923
Fe	106	0.767945	0.825869	0.134127
Fe	107	0.767945	0.741316	0.290725
Fe	108	0.767945	0.656763	0.447323

H_2O adsorption

Element	Atom number	Fractional coordinates of atoms u	v	w
H	1	0.304455	0.153890	0.019177
H	2	0.304455	0.069337	0.175775
H	3	0.304455	0.984785	0.332373
H	4	0.054455	0.811151	0.046079
H	5	0.054455	0.726598	0.202676
H	6	0.054455	0.642045	0.359274
H	7	0.195545	0.268874	0.124377
H	8	0.195545	0.184321	0.280975
H	9	0.195545	0.099768	0.437573
H	10	0.445545	0.611614	0.097476

H	11	0.445545	0.527061	0.254074
H	12	0.445545	0.442508	0.410671
H	13	0.804455	0.153890	0.019177
H	14	0.804455	0.069337	0.175775
H	15	0.804455	0.984785	0.332373
H	16	0.554455	0.811151	0.046079
H	17	0.554455	0.726598	0.202676
H	18	0.554455	0.642045	0.359274
H	19	0.695545	0.268874	0.124377
H	20	0.695545	0.184321	0.280975
H	21	0.695545	0.099768	0.437573
H	22	0.945545	0.611614	0.097476
H	23	0.945545	0.527061	0.254074
H	24	0.945545	0.442508	0.410671
H	25	0.911480	0.634148	0.569032
H	26	0.867277	0.781049	0.559685
H	27	0.316066	0.124099	0.550374
H	28	0.222880	0.235029	0.532639
H	29	0.818144	0.250629	0.547858
H	30	0.769569	0.379976	0.530450
H	31	0.328466	0.428234	0.563350
H	32	0.249636	0.564117	0.569706
H	33	0.350206	0.747286	0.593283
H	34	0.266130	0.872447	0.568378
O	35	0.132800	0.041782	0.000000
O	36	0.132800	0.957229	0.156598
O	37	0.132800	0.872676	0.313195
O	38	0.407756	0.181488	0.023898
O	39	0.407756	0.096935	0.180496
O	40	0.407756	0.012382	0.337093
O	41	0.382800	0.923259	0.065256
O	42	0.382800	0.838706	0.221854

O	43	0.382800	0.754153	0.378451
O	44	0.157756	0.783553	0.041358
O	45	0.157756	0.699000	0.197955
O	46	0.157756	0.614447	0.354553
O	47	0.367200	0.380983	0.143555
O	48	0.367200	0.296430	0.300152
O	49	0.367200	0.211877	0.456750
O	50	0.092244	0.241276	0.119656
O	51	0.092244	0.156723	0.276254
O	52	0.092244	0.072170	0.432852
O	53	0.117200	0.499506	0.078299
O	54	0.117200	0.414953	0.234896
O	55	0.117200	0.330400	0.391494
O	56	0.342244	0.639212	0.102197
O	57	0.342244	0.554659	0.258795
O	58	0.342244	0.470106	0.415392
O	59	0.632800	0.041782	0.000000
O	60	0.632800	0.957229	0.156598
O	61	0.632800	0.872676	0.313195
O	62	0.907756	0.181488	0.023898
O	63	0.907756	0.096935	0.180496
O	64	0.907756	0.012382	0.337093
O	65	0.882800	0.923259	0.065256
O	66	0.882800	0.838706	0.221854
O	67	0.882800	0.754153	0.378451
O	68	0.657756	0.783553	0.041358
O	69	0.657756	0.699000	0.197955
O	70	0.657756	0.614447	0.354553
O	71	0.867200	0.380983	0.143555
O	72	0.867200	0.296430	0.300152
O	73	0.867200	0.211877	0.456750
O	74	0.592244	0.241276	0.119656

O	75	0.592244	0.156723	0.276254
O	76	0.592244	0.072170	0.432852
O	77	0.617200	0.499506	0.078299
O	78	0.617200	0.414953	0.234896
O	79	0.617200	0.330400	0.391494
O	80	0.842244	0.639212	0.102197
O	81	0.842244	0.554659	0.258795
O	82	0.842244	0.470106	0.415392
O	83	0.839209	0.692339	0.547264
O	84	0.231927	0.168830	0.571148
O	85	0.731615	0.305314	0.556392
O	86	0.236041	0.474268	0.557291
O	87	0.280561	0.780263	0.558468
Fe	88	0.482055	0.368145	0.055828
Fe	89	0.482055	0.283592	0.212426
Fe	90	0.482055	0.199039	0.369023
Fe	91	0.232055	0.596896	0.009428
Fe	92	0.232055	0.512343	0.166025
Fe	93	0.232055	0.427790	0.322623
Fe	94	0.017945	0.054619	0.087727
Fe	95	0.017945	0.970067	0.244324
Fe	96	0.017945	0.885514	0.400922
Fe	97	0.267945	0.825869	0.134127
Fe	98	0.267945	0.741316	0.290724
Fe	99	0.267945	0.656763	0.447322
Fe	100	0.982055	0.368145	0.055828
Fe	101	0.982055	0.283592	0.212426
Fe	102	0.982055	0.199039	0.369023
Fe	103	0.732055	0.596896	0.009428
Fe	104	0.732055	0.512343	0.166025
Fe	105	0.732055	0.427790	0.322623
Fe	106	0.517945	0.054619	0.087727

Fe	107	0.517945	0.970067	0.244324
Fe	108	0.517945	0.885514	0.400922
Fe	109	0.767945	0.825869	0.134127
Fe	110	0.767945	0.741316	0.290724
Fe	111	0.767945	0.656763	0.447322

Transition State

Element	Atom number	Fractional coordinates of atoms u	v	w
H	1	0.304455	0.153890	0.019177
H	2	0.304455	0.069337	0.175775
H	3	0.304455	0.984785	0.332373
H	4	0.054455	0.811151	0.046079
H	5	0.054455	0.726598	0.202676
H	6	0.054455	0.642045	0.359274
H	7	0.195545	0.268874	0.124377
H	8	0.195545	0.184321	0.280975
H	9	0.195545	0.099768	0.437573
H	10	0.445545	0.611614	0.097476
H	11	0.445545	0.527061	0.254074
H	12	0.445545	0.442508	0.410671
H	13	0.804455	0.153890	0.019177
H	14	0.804455	0.069337	0.175775
H	15	0.804455	0.984785	0.332373
H	16	0.554455	0.811151	0.046079
H	17	0.554455	0.726598	0.202676
H	18	0.554455	0.642045	0.359274
H	19	0.695545	0.268874	0.124377
H	20	0.695545	0.184321	0.280975
H	21	0.695545	0.099768	0.437573
H	22	0.945545	0.611614	0.097476

H	23	0.945545	0.527061	0.254074
H	24	0.945545	0.442508	0.410671
H	25	0.843379	0.676065	0.526663
H	26	0.758916	0.832615	0.541659
H	27	0.316066	0.124099	0.550374
H	28	0.222880	0.235029	0.532639
H	29	0.818144	0.250629	0.547858
H	30	0.769569	0.379976	0.530450
H	31	0.328466	0.428234	0.563350
H	32	0.249636	0.564117	0.569706
H	33	0.350206	0.747286	0.593283
H	34	0.266130	0.872447	0.568378
O	35	0.132800	0.041782	0.000000
O	36	0.132800	0.957229	0.156598
O	37	0.132800	0.872676	0.313195
O	38	0.407756	0.181488	0.023898
O	39	0.407756	0.096935	0.180496
O	40	0.407756	0.012382	0.337093
O	41	0.382800	0.923259	0.065256
O	42	0.382800	0.838706	0.221854
O	43	0.382800	0.754153	0.378451
O	44	0.157756	0.783553	0.041358
O	45	0.157756	0.699000	0.197955
O	46	0.157756	0.614447	0.354553
O	47	0.367200	0.380983	0.143555
O	48	0.367200	0.296430	0.300152
O	49	0.367200	0.211877	0.456750
O	50	0.092244	0.241276	0.119656
O	51	0.092244	0.156723	0.276254
O	52	0.092244	0.072170	0.432852
O	53	0.117200	0.499506	0.078299
O	54	0.117200	0.414953	0.234896

O	55	0.117200	0.330400	0.391494
O	56	0.342244	0.639212	0.102197
O	57	0.342244	0.554659	0.258795
O	58	0.342244	0.470106	0.415392
O	59	0.632800	0.041782	0.000000
O	60	0.632800	0.957229	0.156598
O	61	0.632800	0.872676	0.313195
O	62	0.907756	0.181488	0.023898
O	63	0.907756	0.096935	0.180496
O	64	0.907756	0.012382	0.337093
O	65	0.882800	0.923259	0.065256
O	66	0.882800	0.838706	0.221854
O	67	0.882800	0.754153	0.378451
O	68	0.657756	0.783553	0.041358
O	69	0.657756	0.699000	0.197955
O	70	0.657756	0.614447	0.354553
O	71	0.867200	0.380983	0.143555
O	72	0.867200	0.296430	0.300152
O	73	0.867200	0.211877	0.456750
O	74	0.592244	0.241276	0.119656
O	75	0.592244	0.156723	0.276254
O	76	0.592244	0.072170	0.432852
O	77	0.617200	0.499506	0.078299
O	78	0.617200	0.414953	0.234896
O	79	0.617200	0.330400	0.391494
O	80	0.842244	0.639212	0.102197
O	81	0.842244	0.554659	0.258795
O	82	0.842244	0.470106	0.415392
O	83	0.723120	0.744467	0.533460
O	84	0.231927	0.168830	0.571148
O	85	0.731615	0.305314	0.556392
O	86	0.236041	0.474268	0.557291

O	87	0.280561	0.780263	0.558468
Fe	88	0.482055	0.368145	0.055828
Fe	89	0.482055	0.283592	0.212426
Fe	90	0.482055	0.199039	0.369023
Fe	91	0.232055	0.596896	0.009428
Fe	92	0.232055	0.512343	0.166025
Fe	93	0.232055	0.427790	0.322623
Fe	94	0.017945	0.054619	0.087727
Fe	95	0.017945	0.970067	0.244324
Fe	96	0.017945	0.885514	0.400922
Fe	97	0.267945	0.825869	0.134127
Fe	98	0.267945	0.741316	0.290724
Fe	99	0.267945	0.656763	0.447322
Fe	100	0.982055	0.368145	0.055828
Fe	101	0.982055	0.283592	0.212426
Fe	102	0.982055	0.199039	0.369023
Fe	103	0.732055	0.596896	0.009428
Fe	104	0.732055	0.512343	0.166025
Fe	105	0.732055	0.427790	0.322623
Fe	106	0.517945	0.054619	0.087727
Fe	107	0.517945	0.970067	0.244324
Fe	108	0.517945	0.885514	0.400922
Fe	109	0.767945	0.825869	0.134127
Fe	110	0.767945	0.741316	0.290724
Fe	111	0.767945	0.656763	0.447322

Hydrolyzation state

Element	Atom number	Fractional coordinates of atoms u	v	w
H	1	0.304455	0.153890	0.019177
H	2	0.304455	0.069337	0.175775

H	3	0.304455	0.984785	0.332373
H	4	0.054455	0.811151	0.046079
H	5	0.054455	0.726598	0.202676
H	6	0.054455	0.642045	0.359274
H	7	0.195545	0.268874	0.124377
H	8	0.195545	0.184321	0.280975
H	9	0.195545	0.099768	0.437573
H	10	0.445545	0.611614	0.097476
H	11	0.445545	0.527061	0.254074
H	12	0.445545	0.442508	0.410671
H	13	0.804455	0.153890	0.019177
H	14	0.804455	0.069337	0.175775
H	15	0.804455	0.984785	0.332373
H	16	0.554455	0.811151	0.046079
H	17	0.554455	0.726598	0.202676
H	18	0.554455	0.642045	0.359274
H	19	0.695545	0.268874	0.124377
H	20	0.695545	0.184321	0.280975
H	21	0.695545	0.099768	0.437573
H	22	0.945545	0.611614	0.097476
H	23	0.945545	0.527061	0.254074
H	24	0.945545	0.442508	0.410671
H	25	0.854215	0.682373	0.516339
H	26	0.663149	0.841246	0.508470
H	27	0.316066	0.124099	0.550374
H	28	0.222880	0.235029	0.532639
H	29	0.818144	0.250629	0.547858
H	30	0.769569	0.379976	0.530450
H	31	0.328466	0.428234	0.563350
H	32	0.249636	0.564117	0.569706
H	33	0.350206	0.747286	0.593283
H	34	0.266130	0.872447	0.568378

O	35	0.132800	0.041782	0.000000
O	36	0.132800	0.957229	0.156598
O	37	0.132800	0.872676	0.313195
O	38	0.407756	0.181488	0.023898
O	39	0.407756	0.096935	0.180496
O	40	0.407756	0.012382	0.337093
O	41	0.382800	0.923259	0.065256
O	42	0.382800	0.838706	0.221854
O	43	0.382800	0.754153	0.378451
O	44	0.157756	0.783553	0.041358
O	45	0.157756	0.699000	0.197955
O	46	0.157756	0.614447	0.354553
O	47	0.367200	0.380983	0.143555
O	48	0.367200	0.296430	0.300152
O	49	0.367200	0.211877	0.456750
O	50	0.092244	0.241276	0.119656
O	51	0.092244	0.156723	0.276254
O	52	0.092244	0.072170	0.432852
O	53	0.117200	0.499506	0.078299
O	54	0.117200	0.414953	0.234896
O	55	0.117200	0.330400	0.391494
O	56	0.342244	0.639212	0.102197
O	57	0.342244	0.554659	0.258795
O	58	0.342244	0.470106	0.415392
O	59	0.632800	0.041782	0.000000
O	60	0.632800	0.957229	0.156598
O	61	0.632800	0.872676	0.313195
O	62	0.907756	0.181488	0.023898
O	63	0.907756	0.096935	0.180496
O	64	0.907756	0.012382	0.337093
O	65	0.882800	0.923259	0.065256
O	66	0.882800	0.838706	0.221854

O	67	0.882800	0.754153	0.378451
O	68	0.657756	0.783553	0.041358
O	69	0.657756	0.699000	0.197955
O	70	0.657756	0.614447	0.354553
O	71	0.867200	0.380983	0.143555
O	72	0.867200	0.296430	0.300152
O	73	0.867200	0.211877	0.456750
O	74	0.592244	0.241276	0.119656
O	75	0.592244	0.156723	0.276254
O	76	0.592244	0.072170	0.432852
O	77	0.617200	0.499506	0.078299
O	78	0.617200	0.414953	0.234896
O	79	0.617200	0.330400	0.391494
O	80	0.842244	0.639212	0.102197
O	81	0.842244	0.554659	0.258795
O	82	0.842244	0.470106	0.415392
O	83	0.625777	0.784547	0.470006
O	84	0.231927	0.168830	0.571148
O	85	0.731615	0.305314	0.556392
O	86	0.236041	0.474268	0.557291
O	87	0.280561	0.780263	0.558468
Fe	88	0.482055	0.368145	0.055828
Fe	89	0.482055	0.283592	0.212426
Fe	90	0.482055	0.199039	0.369023
Fe	91	0.232055	0.596896	0.009428
Fe	92	0.232055	0.512343	0.166025
Fe	93	0.232055	0.427790	0.322623
Fe	94	0.017945	0.054619	0.087727
Fe	95	0.017945	0.970067	0.244324
Fe	96	0.017945	0.885514	0.400922
Fe	97	0.267945	0.825869	0.134127
Fe	98	0.267945	0.741316	0.290724

Fe	99	0.267945	0.656763	0.447322
Fe	100	0.982055	0.368145	0.055828
Fe	101	0.982055	0.283592	0.212426
Fe	102	0.982055	0.199039	0.369023
Fe	103	0.732055	0.596896	0.009428
Fe	104	0.732055	0.512343	0.166025
Fe	105	0.732055	0.427790	0.322623
Fe	106	0.517945	0.054619	0.087727
Fe	107	0.517945	0.970067	0.244324
Fe	108	0.517945	0.885514	0.400922
Fe	109	0.767945	0.825869	0.134127
Fe	110	0.767945	0.741316	0.290724
Fe	111	0.767945	0.656763	0.447322

Release hydronium

Element	Atom number	Fractional coordinates of atoms u	v	w
H	1	0.304455	0.15389	0.019177
H	2	0.304455	0.069337	0.175775
H	3	0.304455	0.984784	0.332373
H	4	0.054455	0.811151	0.046079
H	5	0.054455	0.726598	0.202677
H	6	0.054455	0.642045	0.359274
H	7	0.195545	0.268874	0.124378
H	8	0.195545	0.184321	0.280976
H	9	0.195545	0.099768	0.437573
H	10	0.445545	0.611614	0.097476
H	11	0.445545	0.527061	0.254074
H	12	0.445545	0.442508	0.410672
H	13	0.804455	0.15389	0.019177
H	14	0.804455	0.069337	0.175775

H	15	0.804455	0.984784	0.332373
H	16	0.554455	0.811151	0.046079
H	17	0.554455	0.726598	0.202677
H	18	0.554455	0.642045	0.359274
H	19	0.695545	0.268874	0.124378
H	20	0.695545	0.184321	0.280976
H	21	0.695545	0.099768	0.437573
H	22	0.945545	0.611614	0.097476
H	23	0.945545	0.527061	0.254074
H	24	0.945545	0.442508	0.410672
H	25	0.661052	0.846538	0.508869
H	26	0.316066	0.124099	0.550375
H	27	0.22288	0.235028	0.53264
H	28	0.328466	0.428234	0.563351
H	29	0.249636	0.564117	0.569707
H	30	0.350206	0.747286	0.593285
H	31	0.26613	0.872447	0.568379
O	32	0.1328	0.041782	0
O	33	0.1328	0.957229	0.156598
O	34	0.1328	0.872676	0.313196
O	35	0.407756	0.181488	0.023898
O	36	0.407756	0.096935	0.180496
O	37	0.407756	0.012382	0.337094
O	38	0.3828	0.923259	0.065256
O	39	0.3828	0.838706	0.221854
O	40	0.3828	0.754153	0.378452
O	41	0.157756	0.783553	0.041358
O	42	0.157756	0.699	0.197956
O	43	0.157756	0.614447	0.354554
O	44	0.3672	0.380983	0.143555
O	45	0.3672	0.29643	0.300153
O	46	0.3672	0.211877	0.456751

O	47	0.092244	0.241276	0.119657
O	48	0.092244	0.156723	0.276255
O	49	0.092244	0.07217	0.432852
O	50	0.1172	0.499506	0.078299
O	51	0.1172	0.414953	0.234897
O	52	0.1172	0.3304	0.391495
O	53	0.342244	0.639212	0.102197
O	54	0.342244	0.554659	0.258795
O	55	0.342244	0.470106	0.415393
O	56	0.6328	0.041782	0
O	57	0.6328	0.957229	0.156598
O	58	0.6328	0.872676	0.313196
O	59	0.907756	0.181488	0.023898
O	60	0.907756	0.096935	0.180496
O	61	0.907756	0.012382	0.337094
O	62	0.8828	0.923259	0.065256
O	63	0.8828	0.838706	0.221854
O	64	0.8828	0.754153	0.378452
O	65	0.657756	0.783553	0.041358
O	66	0.657756	0.699	0.197956
O	67	0.657756	0.614447	0.354554
O	68	0.8672	0.380983	0.143555
O	69	0.8672	0.29643	0.300153
O	70	0.8672	0.211877	0.456751
O	71	0.592244	0.241276	0.119657
O	72	0.592244	0.156723	0.276255
O	73	0.592244	0.07217	0.432852
O	74	0.6172	0.499506	0.078299
O	75	0.6172	0.414953	0.234897
O	76	0.6172	0.3304	0.391495
O	77	0.842244	0.639212	0.102197
O	78	0.842244	0.554659	0.258795

O	79	0.842244	0.470106	0.415393
O	80	0.626635	0.78488	0.47191
O	81	0.231927	0.16883	0.571149
O	82	0.236041	0.474267	0.557292
O	83	0.280561	0.780262	0.558469
Fe	84	0.482055	0.368145	0.055828
Fe	85	0.482055	0.283592	0.212426
Fe	86	0.482055	0.199039	0.369024
Fe	87	0.232055	0.596896	0.009428
Fe	88	0.232055	0.512343	0.166026
Fe	89	0.232055	0.42779	0.322624
Fe	90	0.017945	0.054619	0.087727
Fe	91	0.017945	0.970066	0.244325
Fe	92	0.017945	0.885514	0.400923
Fe	93	0.267945	0.825869	0.134127
Fe	94	0.267945	0.741316	0.290725
Fe	95	0.267945	0.656763	0.447323
Fe	96	0.982055	0.368145	0.055828
Fe	97	0.982055	0.283592	0.212426
Fe	98	0.982055	0.199039	0.369024
Fe	99	0.732055	0.596896	0.009428
Fe	100	0.732055	0.512343	0.166026
Fe	101	0.732055	0.42779	0.322624
Fe	102	0.517945	0.054619	0.087727
Fe	103	0.517945	0.970066	0.244325
Fe	104	0.517945	0.885514	0.400923
Fe	105	0.767945	0.825869	0.134127
Fe	106	0.767945	0.741316	0.290725
Fe	107	0.767945	0.656763	0.447323

Release hydronium and hydroxide

Element	Atom	Fractional coordinates of atoms		

	number	u	v	w
H	1	0.304455	0.15389	0.019177
H	2	0.304455	0.069337	0.175775
H	3	0.304455	0.984784	0.332373
H	4	0.054455	0.811151	0.046079
H	5	0.054455	0.726598	0.202677
H	6	0.054455	0.642045	0.359274
H	7	0.195545	0.268874	0.124378
H	8	0.195545	0.184321	0.280976
H	9	0.195545	0.099768	0.437573
H	10	0.445545	0.611614	0.097476
H	11	0.445545	0.527061	0.254074
H	12	0.445545	0.442508	0.410672
H	13	0.804455	0.15389	0.019177
H	14	0.804455	0.069337	0.175775
H	15	0.804455	0.984784	0.332373
H	16	0.554455	0.811151	0.046079
H	17	0.554455	0.726598	0.202677
H	18	0.554455	0.642045	0.359274
H	19	0.695545	0.268874	0.124378
H	20	0.695545	0.184321	0.280976
H	21	0.695545	0.099768	0.437573
H	22	0.945545	0.611614	0.097476
H	23	0.945545	0.527061	0.254074
H	24	0.945545	0.442508	0.410672
H	25	0.316066	0.124099	0.550375
H	26	0.22288	0.235028	0.53264
H	27	0.328466	0.428234	0.563351
H	28	0.249636	0.564117	0.569707
H	29	0.350206	0.747286	0.593285
H	30	0.26613	0.872447	0.568379

O	31	0.1328	0.041782	0
O	32	0.1328	0.957229	0.156598
O	33	0.1328	0.872676	0.313196
O	34	0.407756	0.181488	0.023898
O	35	0.407756	0.096935	0.180496
O	36	0.407756	0.012382	0.337094
O	37	0.3828	0.923259	0.065256
O	38	0.3828	0.838706	0.221854
O	39	0.3828	0.754153	0.378452
O	40	0.157756	0.783553	0.041358
O	41	0.157756	0.699	0.197956
O	42	0.157756	0.614447	0.354554
O	43	0.3672	0.380983	0.143555
O	44	0.3672	0.29643	0.300153
O	45	0.3672	0.211877	0.456751
O	46	0.092244	0.241276	0.119657
O	47	0.092244	0.156723	0.276255
O	48	0.092244	0.07217	0.432852
O	49	0.1172	0.499506	0.078299
O	50	0.1172	0.414953	0.234897
O	51	0.1172	0.3304	0.391495
O	52	0.342244	0.639212	0.102197
O	53	0.342244	0.554659	0.258795
O	54	0.342244	0.470106	0.415393
O	55	0.6328	0.041782	0
O	56	0.6328	0.957229	0.156598
O	57	0.6328	0.872676	0.313196
O	58	0.907756	0.181488	0.023898
O	59	0.907756	0.096935	0.180496
O	60	0.907756	0.012382	0.337094
O	61	0.8828	0.923259	0.065256
O	62	0.8828	0.838706	0.221854

O	63	0.8828	0.754153	0.378452
O	64	0.657756	0.783553	0.041358
O	65	0.657756	0.699	0.197956
O	66	0.657756	0.614447	0.354554
O	67	0.8672	0.380983	0.143555
O	68	0.8672	0.29643	0.300153
O	69	0.8672	0.211877	0.456751
O	70	0.592244	0.241276	0.119657
O	71	0.592244	0.156723	0.276255
O	72	0.592244	0.07217	0.432852
O	73	0.6172	0.499506	0.078299
O	74	0.6172	0.414953	0.234897
O	75	0.6172	0.3304	0.391495
O	76	0.842244	0.639212	0.102197
O	77	0.842244	0.554659	0.258795
O	78	0.842244	0.470106	0.415393
O	79	0.231927	0.16883	0.571149
O	80	0.236041	0.474267	0.557292
O	81	0.280561	0.780262	0.558469
Fe	82	0.482055	0.368145	0.055828
Fe	83	0.482055	0.283592	0.212426
Fe	84	0.482055	0.199039	0.369024
Fe	85	0.232055	0.596896	0.009428
Fe	86	0.232055	0.512343	0.166026
Fe	87	0.232055	0.42779	0.322624
Fe	88	0.017945	0.054619	0.087727
Fe	89	0.017945	0.970066	0.244325
Fe	90	0.017945	0.885514	0.400923
Fe	91	0.267945	0.825869	0.134127
Fe	92	0.267945	0.741316	0.290725
Fe	93	0.267945	0.656763	0.447323
Fe	94	0.982055	0.368145	0.055828

Fe	95	0.982055	0.283592	0.212426
Fe	96	0.982055	0.199039	0.369024
Fe	97	0.732055	0.596896	0.009428
Fe	98	0.732055	0.512343	0.166026
Fe	99	0.732055	0.42779	0.322624
Fe	100	0.517945	0.054619	0.087727
Fe	101	0.517945	0.970066	0.244325
Fe	102	0.517945	0.885514	0.400923
Fe	103	0.767945	0.825869	0.134127
Fe	104	0.767945	0.741316	0.290725
Fe	105	0.767945	0.656763	0.447323

Supplementary Note 1

The thermodynamic voltage of WD onset, which is indeed 0.83 V is only possible under controlled thermodynamic conditions such as 1 M $OH^-$and 1 M H^+ concentration at the AEL and CEL surfaces of the bipolar membranes. However, the results were not reliable enough for comparison of multiple types of membranes with different WD rates (producing $OH^-$and H^+ to varying rates in this work) due to difficulties in maintaining a constant acid-base concentration during the entire WD testing time. Therefore, we have chosen easy to control and reliable testing conditions such as 0.5 M NaCl on both sides of the bipolar membranes, and continuous mixing was done to maintain a neutrality condition of the test solutions during the whole testing time. The pH besides the bipolar membranes was continuously checked using pH strips. Therefore, the WD voltages in this work ar of kinetic in nature which is not dependent on theoretical WD voltage value. Oener et al. have also proved that the WD is pH-dependent and may initiate at voltage < 0.83 depending on the neutrality conditions of the test solutions. Herein, the voltage values at the limiting current density (U_{LCD}, the value at the inflection point of the I-V curves from horizontal to the vertical direction) and the voltage drop at 100 mA cm^{-2} (U_{100}) are taken for a comparison of all the fabricated and commercial bipolar membranes.

Supplementary Discussion

SEM-EDX analysis confirm QPPO penetration within the SCJ

Energy-dispersive X-ray spectroscopy (EDX) in conjunction with scanning electron microscopy (SEM) is conducted to better confirm the QPPO penetration within the junction. The SEM-EDX mapping of fluorine (F, a representative element of Nafion-CEL), bromine (Br, a representative element of QPPO-AEL), carbon (C), and oxygen (O) are shown in Supplementary Fig. 5a. In contrast to a very uniform distribution of F at the interface of C/AEL, a non-uniform impregnating distribution of Br is an indication of QPPO penetration into the junction which is up to the Nafion-CEL surface. Unfortunately, we could not get the mapping of Fe in the junction or within the Nafion during repeated SEM-EDX tests due to its same energy dispersion band

as F (Supplementary Fig. 5b). Moreover, we could also not get the mapping of the nitrogen element (N, a representative element of PANI) except its appearance in Supplementary Fig. 5b due to its difficult detection within the junction and its weak signal. Therefore, we have chosen C (which is more concentrated in PANI) and O as a representative element of the $Fe^{+3}O(OH)$ catalyst. The presence of excessive C and O elements at the interface are predicting the PANI and the $Fe^{+3}O(OH)$ catalyst. Moreover, the excessive presence of Fe in the graph of the elemental composition is an indication of the catalyst presence.

Effect of conductive polyaniline shields in WD activity of the SCBMs

To support the effect of conductive polyaniline shields in WD activity of the shielded catalytic bipolar membranes (SCBMs), we have *in-situ* grown polyaniline on Nafion-CEL in its two states such as conductive emeraldine salt (Nafion_PANI-ES) and non-/less conductive emeraldine base (Nafion_PANI-EB). Both states are chemically confirmed using the FTIR-ATR technique (Supplementary Fig. a). Besides the two characteristic peaks of PANI at 1503 cm^{-1} (benzenoid) and 1599 cm^{-1} (quinonoid), large IR absorption at 1152 cm^{-1} which associates with the vibration mode of an electronic band (N=Q=N, Q refers to the quinonic type rings) indicates the electrically conductive characteristics of the Nafion_PANI-ES surface layer. On the other hand, suppression of the peak at 1152 cm^{-1} in the spectrum of Nafion_PANI-EB predicts decreased conductivity. The observed difference in the conductivities was further evaluated quantitatively using a two-probe resistance testing meter and found a huge difference in the conductivities of both the PANI surface layers as shown in Supplementary Fig. 13b.

Considering the conductivity difference of 1.021 mS cm^{-1} (Nafion_PANI-ES: 1.049 mS cm^{-1} and Nafion_PANI-EB: 28.558 μS cm^{-1}), we have fabricated two types of bipolar membranes comprising the conductive polyaniline shield (SBMs) and non-conductive polyaniline shield (NSBMs). After saturation in the test solution, we have recorded the polarization I-V curves (Supplementary Fig. 13c). Interestingly, we not only found a very large transmembrane potential drop in NSBMs but also observed a continuously increasing potential lag at the same current densities than the SBMs owing to less conductive characteristics.

Moreover, the SBMs are exhibiting a near-vertical trend in the I-V curve (Transmembrane potential drop, $V_{SM} \rightarrow 0$), whereas the I-V curve of NSMs inclining toward the horizontal axis ($V_{NSM} \rightarrow \infty$). This difference is possibly due to the conductive characteristics of the PANI-ES in SMs and non-/less conductivity of the PANI-EB in NSMs. Thus, the conductive shields are beneficial for enhancing WD performance and stability.

EIS analysis of the SCBMs at low current densities

Electrochemical impedance spectroscopy (EIS) measurements under galvanostatic mode (Supplementary Fig. 8a) at very low current densities (1 and 3 mA cm^{-2}) can elucidate complex ionic transportation and water dissociation resistances taking place within the shielded catalytic bipolar membranes (SCBMs). Effect of the interfaces at the C/AEL, the polyaniline shields, and the catalyst can also be predicted quantitatively by fitting an equivalent electrical circuit (EEC) model, $R_{C+A+BLs}(Q_{CJI}(R_{CJI}(Q_{PCI}R_{PCI})(Q_{JAI}(R_{JAI}))))G$, and simulating the EIS data using electrochemical analysis software (ZSimpWin, PAR Inc. USA). A simulated $R_{C+A+BLs}$: 9.493 ± 0.05 Ω cm^2 value specifies cumulative resistance of the CEL, AEL, and boundary layers of the SCBMs at 1 mA cm^{-2} which was observed to be decreased at 3 mA cm^{-2} (8.647 ± 0.05 Ω cm^2). This decrease in resistance at comparatively large applied current density is beneficial to enhance the diffusion of the feed water.

The change in porosity/inhomogeneity of the shielded catalytic junction beginning from the CEL-junction-interface (CJI) containing the pre-PANI shield, PANI-catalyst-interface (PCI) containing the *in-situ* produced shielded goethite $Fe^{+3}O(OH)$ catalyst, and junction-AEL-interface (JAI) comprising the post-PANI shield is correlated by constant phase elements (Q) where the Q index ($0 < Q\text{-}n < 1$) is believed to be inversely related to the porosity/inhomogeneity. A large value of $Q\text{-}n_{CJI}$ (0.726), smaller $Q\text{-}n_{PCI}$ (0.603), and largest $Q\text{-}n_{JAI}$ (0.873) is indicative of porous/inhomogeneous morphology of the junction which becomes denser towards the adjacent C/AEL (Supplementary Table 5). Moreover, a comparatively smallest resistance (R_{CJI}: 1.961 Ω cm^2, R_{PCI}: 1.293 Ω cm^2, and R_{JAI}: 1.779 Ω cm^2) and largest admittance ($Q\text{-}Y_{CJI}$: 0.002 S cm^{-2} s^n, $Q\text{-}Y_{PCI}$: 0.043 S cm^{-2} s^n, and $Q\text{-}Y_{JAI}$: 1.592E-4 S cm^{-2} s^n) of the PCI at both the current densities predict fast ionic

transportation and WD within the junction due to abundance of PANI and the catalyst. The increased value of the Gerischer element (G) is further describing the fast water dissociation reaction rate at increased current density (Supplementary Table 5). Briefly, the EIS measurements, the EEC modelling, and simulation of the EIS data predict porous morphology and small charge transfer or WD reaction resistance at the innermost section of the shielded catalytic junction which changes inversely when moving away from the junction, towards the junction-C/AEL-interfaces and the adjacent cation-anion exchange layers.

Mechanism of the WD with goethite $Fe^{+3}O(OH)$ catalyst

Previously, the mechanism of WD catalytic reactions for junctional catalyst has never been thoroughly studied by others in their work of bipolar membranes. However, we hereby propose a possible water dissociation mechanism for our *in-situ* produced goethite $Fe^{+3}O(OH)$ as a junctional catalyst in SCBMs. We performed the theoretical calculations and tried to explain the water dissociation mechanism in a simplistic way for bipolar membrane scientists as follows. We added five water molecules on the model of $Fe^{+3}O(OH)$ surface, one of which was dissociated, and the remaining four were regarded as the explicit solvent. The illustrations matching the DFT calculations indicate that the electron-rich oxygen in the water molecules and the electron-deficient iron on the catalyst surface attract each other, causing the water molecules to absorb on the catalyst surface. The second nearby iron infects the electron-rich oxygen and pulls it while keeping the hydrogen at its original position of 1st iron atom. The function of both the iron atoms decreases the electron density of oxygen, lowers the strength by increasing the length of the H−OH bond until dissociates the water molecule into H^+ and OH^-. The produced protons and hydroxides leave the surface of the catalyst, permeates through the adjacent C/AEL in opposite directions, and enter the corresponding acid-base compartments as shown in Fig. 3b and Fig. 6b, respectively. We found that the *in-situ* produced goethite $Fe^{+3}O(OH)$ junctional catalyst lowers the activation energy barrier from 5.15 (uncatalyzed WD) to only 1.06 eV per HO−H bond, provide stable WD in SCBMs, and avoid the delamination of C/AEL. In comparison with other well-known non-junctional catalysts such as Pt, Fe-MOS_2, NiFe-LDH, MoO_2, ZnCdS, Cu, and MoS_2 (Fig. 3c) also shows that the goethite $Fe^{+3}O(OH)$ in this work can reach the average water dissociation performance.

Supplementary References

1 Xiao, Y. & Cho, C. Experimental Investigation and Discussion on the Mechanical Endurance Limit of Nafion Membrane Used in Proton Exchange Membrane Fuel Cell. *Energies* **7**, 6401-6411, (2014).

2 Biesinger, M. C. *et al.* Resolving surface chemical states in XPS analysis of first row transition metals, oxides and hydroxides: Cr, Mn, Fe, Co and Ni. *Appl. Surf. Sci.*, **257**, 2717-2730, (2011).

3 Yan, Z. *et al.* The balance of electric field and interfacial catalysis in promoting water dissociation in bipolar membranes. *Energy Environ. Sci.* **11**, 2235-2245, (2018).

4 Zabolotskii, V., Sheldeshov, N. & Melnikov, S. Heterogeneous bipolar membranes and their application in electrodialysis. *Desalination* **342**, 183-203, (2014).

5 Onsager, L. Deviations from Ohm's law in weak electrolytes. *J. Chem. Phys.* **2**, 599-615 (1934).

6 Mareev, S. A. *et al.* A comprehensive mathematical model of water splitting in bipolar membranes: Impact of the spatial distribution of fixed charges and catalyst at bipolar junction. *J. Membr. Sci.* **603**, (2020).

7 Wrubel, J. A., Chen, Y., Ma, Z. & Deutsch, T. G. Modeling Water Electrolysis in Bipolar Membranes. *J. Electrochem. Soc.*, 167, 114502 (2020).

8 Shen, C., Wycisk, R. & Pintauro, P. N. High performance electrospun bipolar membrane with a 3D junction. *Energy Environ. Sci.* **10**, 1435-1442, (2017).

9 Wang, A., Peng, S., Wu, Y., Huang, C. & Xu, T. A hybrid bipolar membrane. *J. Membr. Sci.* **365**, 269-275, (2010).

10 Xue, Y., Wang, N., Huang, C., Cheng, Y. & Xu, T. Catalytic water dissociation at the intermediate layer of a bipolar membrane: The role of carboxylated Boltorn® H30. *J. Membr. Sci.* **344**, 129-135, (2009).

11 Rajesh, A. M., Chakrabarty, T., Prakash, S. & Shahi, V. K. Effects of metal alkoxides on electro-assisted water dissociation across bipolar membranes. *Electrochim. Acta* **66**, 325-331, (2012).

12 Abdu, S. *et al.* Catalytic polyelectrolyte multilayers at the bipolar membrane interface. *ACS Appl. Mater. Interfaces* **5**, 10445-10455, (2013).

13 McDonald, M. B., Freund, M. S. & Hammond, P. T. Catalytic, Conductive Bipolar Membrane Interfaces through Layer-by-Layer Deposition for the Design of Membrane-Integrated Artificial Photosynthesis Systems. *ChemSusChem* **10**, 4599-4609, (2017).

14 Liu, X. *et al.* A BiOCl/bipolar membrane as a separator for regenerating NaOH in water-splitting cells. *RSC Adv.* **6**, 9880-9883, (2016).

15 Wang, Q., Wu, B., Jiang, C., Wang, Y. & Xu, T. Improving the water dissociation efficiency in a bipolar membrane with amino-functionalized MIL-101. *J. Membr. Sci.* **524**, 370-376, (2017).

16 Zhu, W. *et al.* Rapid spray-crosslinked assembly of a stable high-performance polyelectrolyte bipolar membrane. *RSC Adv.* **7**, 36313-36318, (2017).

17 Manohar, M., Shukla, G., Pandey, R. P. & Shahi, V. K. Efficient bipolar membrane with protein interfacial layer for optimal water splitting. *J. Ind. Eng. Chem.* **47**, 141-149, (2017).

18 Pan, J. F. *et al.* Preparation of bipolar membranes by electrospinning. *Mater. Chem. Phys.* **186**, 484-491, (2017).

19 Manohar, M., Das, A. K. & Shahi, V. K. Efficient Bipolar Membrane with Functionalized Graphene Oxide Interfacial Layer for Water Splitting and Converting Salt into Acid/Base by Electrodialysis. *Ind. Eng. Chem. Res.* **57**, 1129-1136, (2018).

20 Kumar, M. & Shahi, V. K. Heterogeneous–homogeneous composite bipolar membrane for the conversion of salt of homologous carboxylates into their corresponding acids and bases. *J. Membr. Sci.* **349**, 130-137, (2010).

21 Liu, X., Jian, X., Yang, H., Song, X. & Liang, Z. A photocatalytic graphene quantum dots–Cu2O/bipolar membrane as a separator for water splitting. *New J. Chem.* **40**, 3075-3079, (2016).

22 Zhou, T.-j. *et al.* Preparation and characterization of bipolar membranes modified by photocatalyst nano-ZnO and nano-CeO2. *Appl. Surf. Sci.* **258**, 4023-4027, (2012).
