# Supplementary material for: Shielded goethite catalyst that enables fast water dissociation in bipolar membranes
Source: Nat Commun. 2021 Jan 4;12:9. doi: 10.1038/s41467-020-20131-1 (PMC7782813; doi:10.1038/s41467-020-20131-1)
Supplement: Supplementary file 2 — Description of Additional Supplementary Files [file 41467_2020_20131_MOESM2_ESM.pdf]

Description of Additional Supplementary Files

File name: Supplementary Data 1

Description: Contains all the .cif files that are used to perform the DFT calculations in Figure 3c and Supplementary Fig. 18b.

File name: Supplementary Data 2

Description: Contains one .cif file of the synthesized goethite $Fe^{+3}O(OH)$ catalyst (crystal structure is exhibited as Inset of Fig. 2g).
